# C_60_ Fullerene as the Active Site for CO_2_ Electroreduction

**DOI:** 10.1002/anie.202511924

**Published:** 2025-07-25

**Authors:** Si‐Wei Ying, Yuhang Wang, Peng Du, Qiang Wang, Changming Yue, Di Zhang, Zuo‐Chang Chen, Jian‐Wei Zheng, Su‐Yuan Xie, Hao Li

**Affiliations:** ^1^ Advanced Institute for Materials Research (WPI‐AIMR) Tohoku University Sendai 980‐8577 Japan; ^2^ State Key Laboratory of Physical Chemistry of Solid Surfaces iChEM (Collaborative Innovation Center of Chemistry for Energy Materials) College of Chemistry and Chemical Engineering Xiamen University Xiamen 361005 China; ^3^ State Key Laboratory of Coal Conversion Institute of Coal Chemistry Chinese Academy of Sciences Taiyuan Shanxi 030001 China; ^4^ Department of Physics Southern University of Science and Technology Shenzhen China

**Keywords:** CO_2_RR, Electric field effects, Electrocatalyst, Fullerene (C_60_), pH‐dependent microkinetic modeling

## Abstract

Fullerene (C_60_) was considered as a catalyst promoter in various electrochemical reactions, yet its catalytic role in enhancing catalytic performance beyond electron transfer remains a puzzle to chemists. Traditional simulations imply C_60_’s inertness in CO_2_ reduction reaction (CO_2_RR) due to weak interaction with COOH* intermediates. Here, according to a pH‐field coupled microkinetic model at reversible hydrogen electrode (RHE) scale, we demonstrate that C_60_ acts as molecular active sites to facilitate the CO_2_RR toward CO through a strong binding to COOH* in the electrochemical conditions. This binding is mainly due to the unique structure of C_60_ that induces large dipole moment changes to stabilize COOH* intermediates across different pH conditions. By detailed comparison of experimental CO_2_RR observations and quantitative pH‐dependent modeling, this work provides new insights on C_60_‐based catalysts, highlighting the large dipole moment change upon adsorption at curved surfaces should not be dismissed when analyzing the pH‐dependent binding strength and electrocatalytic activity.

## Graphical abstract

We reveal a striking finding: Fullerene (C_60_), beyond its classical role as an electron buffer, can directly serve as a molecular active site for CO₂ reduction. Through data mining, ab initio simulations, and pH–field coupled microkinetics, we uncover that C_60_’s unique curvature induces strong dipole responses, stabilizing COOH* intermediates and enabling a pH‐dependent volcano. This work redefines the catalytic nature of curved carbon materials.

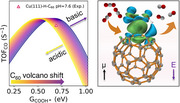

## Introduction

Fullerene (C_60_) materials have been widely recognized for its role as an electronic modulator, altering the electron structures of associated metal catalysts in energy storage and conversion processes.^[^ ^1^, ^2^, ^3^, ^4^, ^5^, ^6^ ^]^ Its unique cage‐like structure and delocalized π‐electron system render its excellent properties of electron buffer,^[^ ^5^ ^]^ enabling efficient charge transfer processes in applications such as hydrogen evolution,^[^ ^7^ ^]^ oxygen reduction,^[^ ^3^ ^]^ and carbon dioxide (CO_2_) reduction reactions.^[^ ^5^, ^8^ ^]^ By integrating fullerene into catalytic systems, performances including reaction rates, selectivity, and durability can be principally improved, opening possibilities for making fullerenes as advanced materials in green technologies.^[^ ^5^, ^9^ ^]^

Electrochemical conversion of CO_2_ to CO holds great promise, as CO is a crucial renewable feedstock for the widely‐applied Fischer–Tropsch process and other industrial applications.^[^ ^10^, ^11^ ^]^ The use of carbon‐based electrocatalysts, such as traditional graphene‐based catalysts and carbon‐coated catalysts, primarily leverages their physical properties to facilitate rapid electron conduction,^[^ ^12^ ^]^ prevent corrosion of metal active sites, and shield against electrochemical oxidation and physical agglomeration.^[^ ^13^ ^]^ However, increasing the applied bias to boost current density at metal active sites will simultaneously promote the competitive hydrogen evolution reaction (HER), which suppresses CO selectivity due to the scaling relationships between COOH* and H* intermediates.^[^ ^14^ ^]^

**Figure 1:**
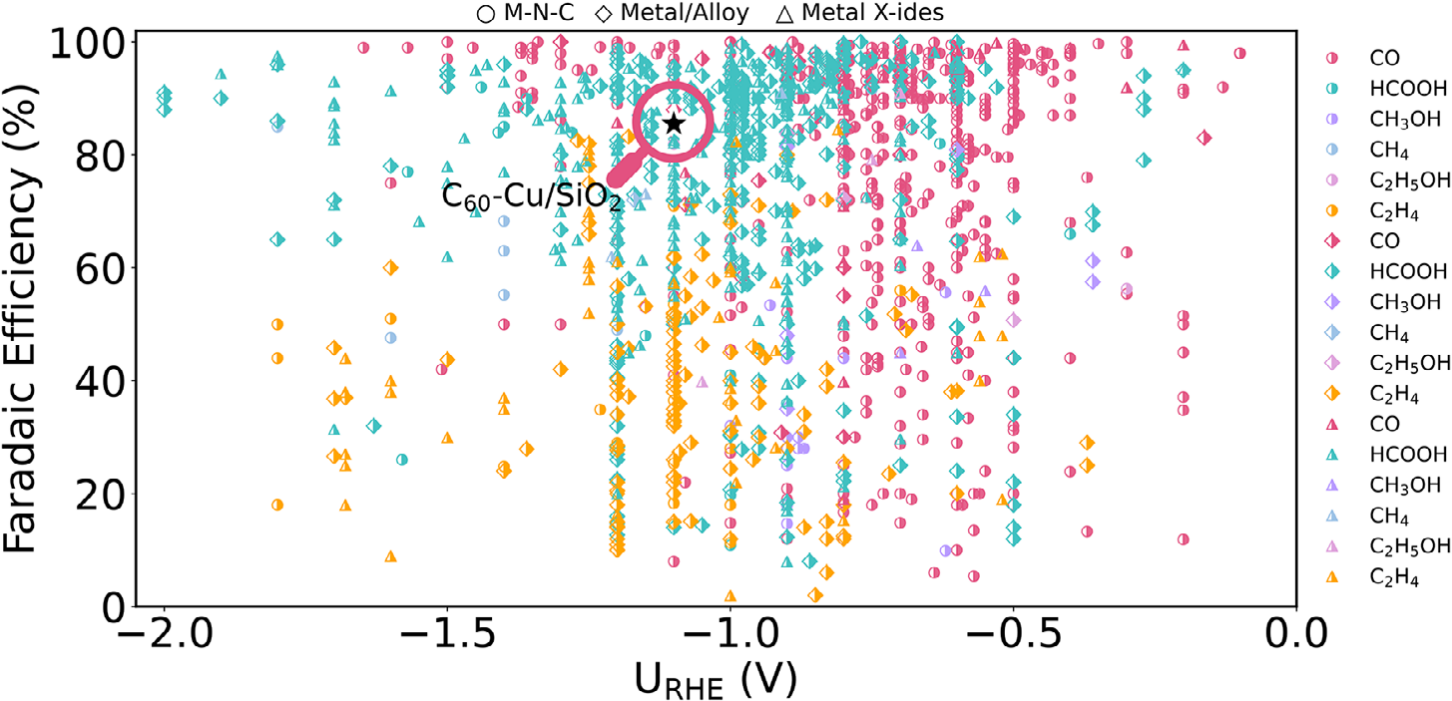
Overview of CO_2_RR activity analysis for 2562 reported catalysts spanning the past decade. Data were extracted from original references and summarized in Table S1. Circles represent products from M─N─C catalysts, diamonds represent products from metal and alloy catalysts, and triangles represent products from metal X‐ide catalysts. A typical C_60_‐based catalyst is denoted by five‐pointed star. The red, green, purple, blue, pink, and yellow colors correspond to the products of CO, HCOOH, CH_3_OH, CH_4_, C_2_H_5_OH, and C_2_H_4_, respectively.

C_60_, with contributions mainly from *s*‐/*p*‐orbital, may effectively bypass the constraints imposed by conventional *d*‐band characteristics of metal catalysts and scaling relationships between COOH* and H* intermediates.^[^ ^14^, ^15^ ^]^ While “electron buffering effects”^[^ ^5^, ^6^, ^16^ ^]^ have recently been exploited in catalysis, C_60_’s role remains to be explored. Through analysis of extensive experimental data via a large‐scale data mining from CO_2_RR literatures published in the past decade (Figure 1), we identified that C_60_ can actively participate in CO_2_RR, directly influencing catalytic performance: even using Cu as the substrate (which conventionally favors multi‐carbon products in CO_2_RR), the inclusion of C_60_ leads to a high Faradaic efficiency (FE) of CO (−90%). This discovery challenges catalytic assumptions about limitation of C_60_ on a promoter and exemplifies the need to reassess its intrinsic role. Despite the simplicity of the two‐electron transfer process in the CO_2_‐to‐CO conversion, the actual reaction mechanisms are complex,^[^ ^17^, ^18^ ^]^ including factors like interfacial charge, solvent effects, applied potential at the reversible hydrogen electrode (RHE) scale, as well as the pH conditions. Accurately capturing these components is challenged to traditional ab initio models.^[^ ^19^ ^]^

The computational hydrogen electrode (CHE) method, widely used in density functional theory (DFT) calculations,^[^ ^20^ ^]^ is a standard approach for modeling electrocatalysis. While sometimes effective in capturing general trends, this method might overlook real the surface states and fails to accurately predict experimental observations, such as current densities (*j*) and Tafel slopes. Moreover, it struggles with different pH conditions, as the traditional H^+^ activity correction term to the reaction energy (*i.e*., 0.059 * pH) shifts in the same way as the potential shifts at the standard hydrogen electrode (SHE) scale, which fails to analyze the pH‐dependent behavior at the RHE scale,^[^ ^21^ ^]^ leading to discrepancies between theoretical and experimental observations. Recent studies have shown that activity in metal‐nitrogen‐carbon (M─N─C) catalysts for CO_2_‐to‐CO conversion depends on key descriptors: CO_2_* and COOH* binding strengths, influenced by narrow *d*‐states and CO_2_* dipoles.^[^ ^22^ ^]^ Although new models incorporating electric field effects and microkinetic analysis have improved understanding of CO_2_RR,^[^ ^23^ ^]^ they are still difficult to achieve accurate predictions of activity and selectivity under complex conditions, for example, considering the electrode acidity,^[^ ^24^ ^]^ consequentially causing deviations between real working conditions and models for CO_2_RR. To address these challenges, it is crucial to understand the atomic‐scale behavior of C_60_ in CO_2_RR, particularly its interactions with key intermediates, rate‐determining steps, and RHE scale's pH‐dependent activity under applied potential.

In this work, we present a comprehensive mechanistic study of CO_2_RR on C_60_‐based catalysts, introducing a robust model that can potentially be extended to other electrocatalytic processes. We reveal that the unique dipole moment and polarizability of C_60_’s curved surface under electric fields plays a crucial role in tuning the binding energies of reaction intermediates, distinctly differing from planar graphene and metal catalysts. On basis of the analysis of the reaction kinetics of CO_2_ activation, COOH* reduction to CO*, and CO* desorption, a pH‐dependent microkinetic activity volcano at the RHE scale was derived using COOH* binding strength as the descriptor (*i.e*., the independent variable). This model enables a direct comparison of competing reaction steps across potential and pH conditions. Furthermore, our finding demonstrate that C_60_‐based catalysts exhibit enhanced activity in alkaline media due to the stabilization of COOH* under electric fields, a direct consequence of the C_60_ molecule's intrinsic curvature‐induced dipole. Such a comprehensive analysis offers new insights into the design of C_60_‐based and carbon‐based electrocatalysts, highlighting the role of surface curvature geometrics in optimizing COOH* binding strength and strategically tuning of reaction conditions to enhance efficiency and selectivity.

## Results and Discussion

### The Role of C_60_

**Figure 2:**
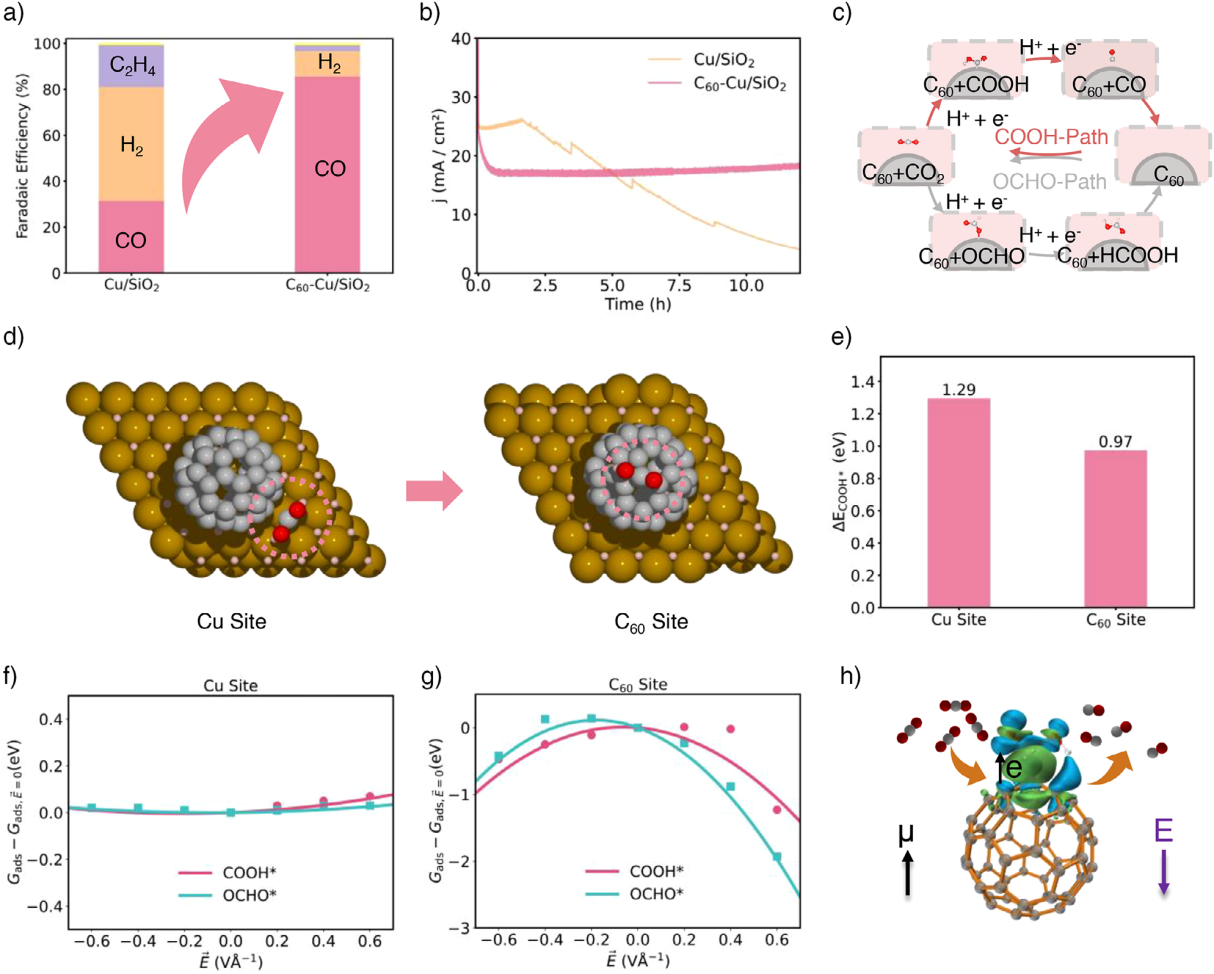
a) Faradaic efficiency (FE) of electrochemical CO_2_RR to CO over Cu/SiO_2_ and C_60_‐Cu/SiO_2_ catalysts. b) Stability comparison of CO_2_RR over Cu/SiO_2_ and C_60_‐Cu/SiO_2_ catalysts at −1.1 V_RHE_. c) Distinct reduction pathways for COOH* and OCHO* intermediates. d) Changes of active sites from Cu surface to C_60_ molecule for the key COOH* adsorbate. e) Relative energy differences between Cu and C_60_ active sites in the absence of an electric field. Gibbs free energy change of COOH* and OCHO* under an applied electric field at f) Cu sites and g) C_60_ sites. h) Charge density difference of COOH* on C_60_, with blue and green isosurfaces representing electron accumulation and depletion, respectively. Atom colors: Brown (Cu), gray (C), red (O), and pink (H).

An initial experiment of CO_2_RR revealed that the Cu/SiO_2_ catalyst, operating at −1.1 V_RHE_, displayed conventional Cu surface activity, producing both CO and C_2_H_4_, but with CO_2_RR competing unfavorably against the HER. However, when C_60_ was integrated with Cu/SiO_2_, the FE of CO rose dramatically to 85.5%, with negligible C_2_H_4_ formation (Figure 2a), signaling a shift in the catalytic active sites. Furthermore, the C_60_‐Cu/SiO_2_ catalyst demonstrated robust stability, with no deactivation or poisoning of the active sites under experimental conditions (Figures 2b and S1). Details of the experimental methods can be found in the Supporting Information.

To understand the observed catalytic performances, Figure 2c illustrates the mechanisms leading to CO and HCOOH production on the electrocatalysts. CO is formed via a two‐electron pathway: COOH* → CO* → CO, while HCOOH is produced through an alternative pathway: OCHO* → HCOOH* → HCOOH. Specifically, the COOH‐pathway can be detailed as: CO_2_ + (H^+^ + e^−^) → COOH*, followed by COOH* + (H^+^ + e^−^) → CO* + H_2_O. In contrast, the OCHO‐pathway proceeds as: CO_2_ + (H^+^ + e^−^) → OCHO*, followed by OCHO* + (H^+^ + e^−^) → HCOOH*. Product analysis confirms that COOH* is the dominant intermediate formed after the first proton‐electron transfer, leading to CO formation.

Figure 2d further illustrates the two distinct active sites within the C_60_‐Cu/SiO_2_ catalyst: the Cu site and the C_60_ site. Simulations of the relative electronic energy of the COOH* intermediate at both sites reveal that C_60_ exhibited a more negative adsorption energy, indicating stronger bonding, which make it more effective at activating CO_2_ (Figure 2e). Further analysis showed that, unlike the Cu site, which remained almost unresponsive to electric fields (Figure 2f), the C_60_ site exhibited a pronounced energy decrease under an applied field, stabilizing COOH* intermediate and significantly enhancing CO_2_ activation (Figure 2g). Differential charge density calculations (Figure 2h) revealed a notable electron flow due to C_60_’s unique spherical structure, effectively activating the asymmetric COOH* intermediate. This characteristic can be a key factor in C_60_’s role as an active site for CO_2_RR. These insights led us to further design a series of model catalysts featuring C_60_ as an active site, systematically exploring changes in intermediate adsorption energies under various electric field to uncover the origin of C_60_’s catalytic activity. This is consistent with the recent study uncovering that C_60_ can combine ammonia intermediates to form C_60_‐NH* and C_60_‐NH_2_*,^[^ ^9^ ^]^ resulting in promoted nitrogen reduction activity.

### The Effect of C_60_ Curved Surfaces and Electric Field

Following the catalytic performance analysis of C_60_‐Cu/SiO_2_ catalyst in CO_2_RR, as summarized in Figures 2 and S2, we delved into the underlying mechanisms that drive the enhanced activity observed in C_60_‐based catalysts. The distinct curvature of C_60_, setting it apart from traditional flat catalysts such as graphene, is hypothesized to play a pivotal role in modulating the adsorption energies of CO_2_RR intermediates under the external electric fields.^[^ ^25^ ^]^ While C_60_ itself possesses a wide bandgap, its integration with graphene—especially defected graphene such as Ox‐Graphene‐C_60_, significantly reduces the bandgap, potentially even conferring metallic properties (Figure S9). This unique combination of materials renders C_60_‐based systems promising as electrocatalytic materials, offering an approach for enhancing catalytic performance. These electronic modifications likely contribute to the improved interactions with intermediates under external electric fields.

**Figure 3:**
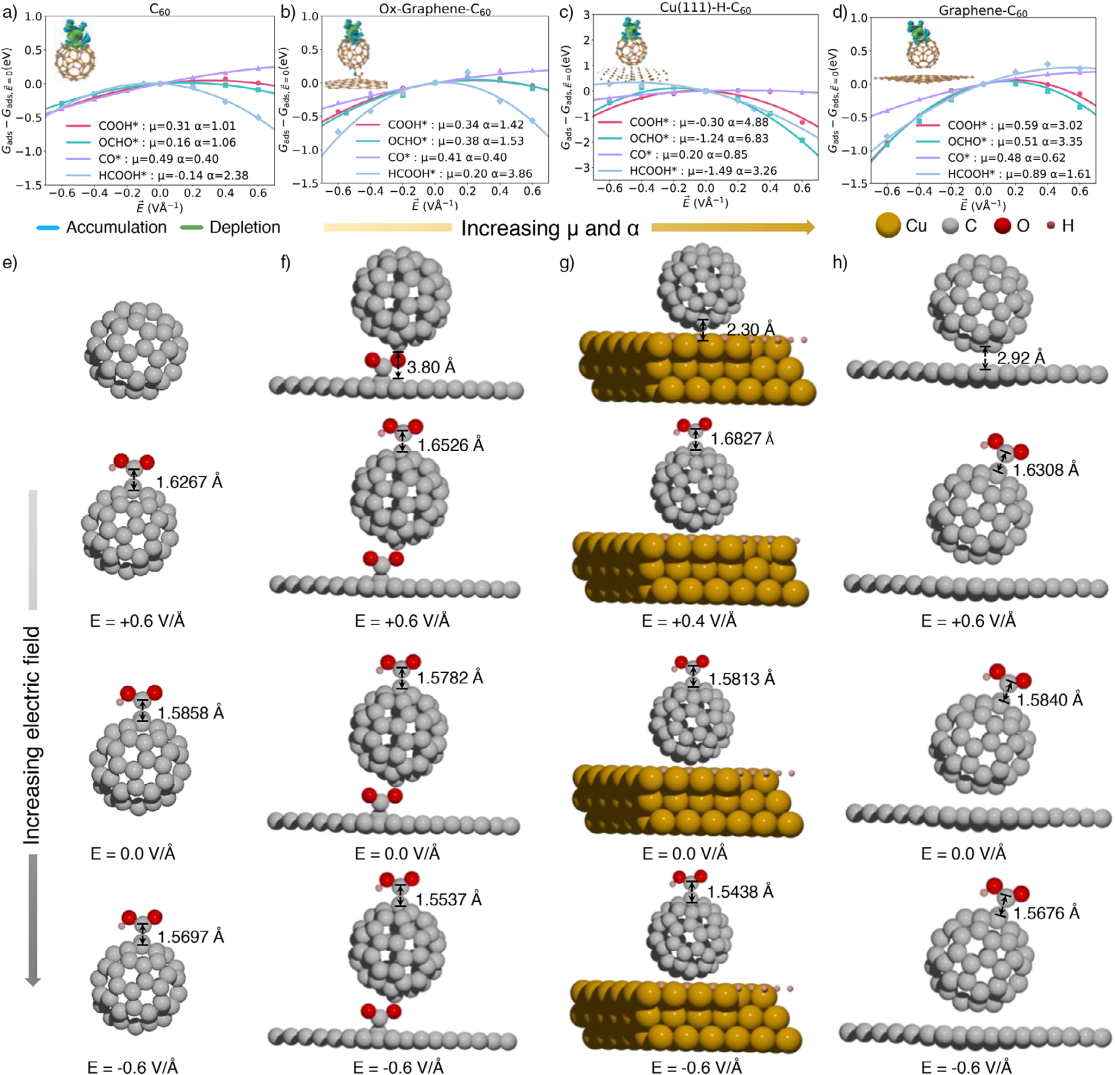
Electric field effects on the adsorption energies of CO_2_RR adsorbates, with fitted values for μ (dipole moment, еÅ) and α (polarizability, e^2^ V^−1^) for a) C_60_, b) Ox‐Graphene‐C_60_, c) Cu (111)‐H‐C_60_, and d) Graphene‐C_60_. Structures and COOH* intermediates under the electric field are shown for e) C_60_, f) Ox‐Graphene‐C_60_, g) Cu(111)‐H‐C_60_, and h) Graphene‐C_60_. Isosurface value: 0.0015 еÅ^−3^.

Figure 3a–d demonstrates how an external electric field impacts the adsorption energies of CO_2_RR intermediates across various C_60_‐based catalysts,^[^ ^26^ ^]^ including C_60_, Ox‐Graphene‐C_60_, Cu(111)‐H‐C_60_, and Graphene‐C_60_. The electric field interacts with intermediates with a substantial dipole moment (μ) and polarizability (α). When comparing the active site on C_60_ to those on H* saturated Cu(111) surfaces,^[^ ^27^ ^]^ the unique curvature and electronic properties of C_60_‐based catalysts under electric fields lead to more negative adsorption energies for COOH* intermediates (Figure S3). Conversely, Cu(111) surfaces and planar graphene show only slight changes in adsorption energies under electric fields (Figures 2f, S4, and S5), with negligible values for μ and α, whereas the C_60_‐based catalysts display substantial improvements.

Negative electric fields increase the μ and α values of adsorbed C_60_‐based catalysts, indicating the adsorption energies significantly benefit from the electric field.^[^ ^21^ ^]^ This substantial polarizability is attributed to the curvature of C_60_,^[^ ^28^ ^]^ which boosts its ability to polarize and stabilize adsorbed intermediates (Figure S6). The curved C_70_ surface has a similar effect (Figure S7). The corresponding enhancement in adsorption energies indicates a strengthened interaction between C_60_ and COOH* intermediate under the electric field. Specifically, in the case of C_60_, the bond length between the carbon atom of C_60_ and the carbon atom of COOH* intermediate shortens from 1.63 Å under a positive electric field to 1.59 Å with no electric field, and further to 1.57 Å under a negative electric field (Figure 3e–h). Under identical electric fields, the more negative ΔG_COOH*_ on Cu(111)‐H‐C_60_ than pristine C_60_ is primarily attributed to enhanced charge transfer at the interface (Figure S8). The isosurfaces provided further detail, illustrating regions of electron accumulation and depletion, emphasizing how the electric field facilitates stronger binding of COOH* by redistributing the charge on catalyst surfaces.^[^ ^17^ ^]^ This charge redistribution lowers the activation energy required for CO_2_RR, thereby enhancing the overall catalytic efficiency of C_60_‐based catalysts.

### The Effects of Potentials of Zero Charge (PZCs) and pH

While the electric field has been proven to impose a notable impact on both COOH* and OCHO* intermediates, the differences in selectivity and activity among C_60_‐based catalysts remains to be fully explained. Additional factors such as potentials of zero charge (PZCs), solvation effects, pH (at the RHE scale), as well as reaction thermodynamics, must be considered for comprehensive evaluation of catalytic performances.^[^ ^29^ ^]^

**Figure 4:**
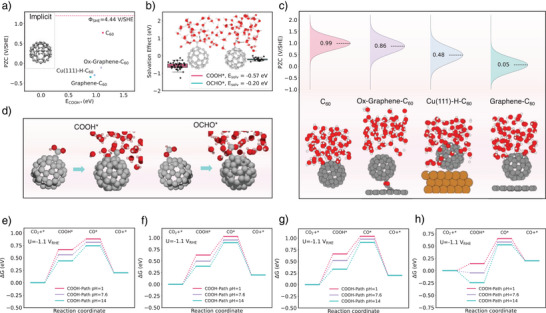
Potentials of zero charge (PZCs) calculated using a) implicit models and c) explicit models. Solvent effects on the b) binding energy changes and d) configuration changes of COOH* and OCHO* in the presence of explicit water molecules. COOH‐Path along with their free energy evolutions on e) C_60_, f) Ox‐Graphene‐C_60_, g) Cu(111)‐H‐C_60_, and h) Graphene‐C_60_ at pH = 1, 7.6, and 14 with U = −1.1 V_RHE_. The normal distributions in c) were driven from over 2000 steps of AIMD simulations of the catalyst‐water interface, while the box plots in b) are based on at least 50 fully optimized distinct surface‐adsorbate configurations for each intermediate.

Figure 4a illustrates that pure C_60_ exhibits a highly positive PZC (+0.77 V), whereas C_60_ catalysts modified with metal or non‐metal components display negative PZC values (−0.10 V for Ox‐Graphene‐C_60_, −0.30 V for Cu(111)‐H‐C_60_, and −0.34 V for Graphene‐C_60_). Explicit model further shows deviations up to 1.0 V/SHE across all C_60_‐based catalysts (Figure 4c), with values of +0.99 V for C_60_, +0.86 V for Ox‐Graphene ‐C_60_, +0.48 V for Cu(111)‐H‐C_60_, and +0.05 V for Graphene‐C_60_. These deviations indicate inherent limitations of implicit model in accurately capturing the PZC when water molecules interact with active sites or surfaces, as shown in Figure S10 and Table S2. The orientation and arrangement of water molecules create a more realistic electrostatic environment, influencing surface charge distribution and thus PZC values.^[^ ^30^ ^]^ To establish a more unified model, we employed an average of PZCs derived from the ab initio molecular dynamics (AIMD) simulations to inform our microkinetic activity analysis.

Solvent effects play a critical role, especially when COOH* and OCHO* intermediates are involved.^[^ ^31^ ^]^ Under the implicit solvent model, the adsorption energy and configuration changes will not be greatly affected by the addition of a water layer, while the explicit model we use can take into account the competitive adsorption of water in AIMD, which is important for the adsorption energy of COOH* and OCHO*. An explicit water layer (thickness: 10 Å, density: *ρ* = 1 g cm^−^^3^) was constructed around the adsorbed COOH* or OCHO* intermediates. Figure 4b shows data through a pool of over 2000 AIMD simulations steps, which sampled more than 50 fully optimized distinct adsorbate‐substrate configurations to estimate solvent effect for each intermediate. COOH* forms hydrogen bonds with two neighboring water molecules, whereas OCHO* is bonded only one. These hydrogen bonding interactions significantly lower the energy of the COOH* intermediate and lead to a shortened bond between COOH* and C_60_, as illustrated in Figure 4d. The mean value of ‐0.57 and −0.20 eV for COOH* and OCHO*, respectively, were incorporated into our model to adjust the adsorption energies accurately.

Thermodynamic analysis revealed that the first hydrogenation step leading to COOH* is energetically more favorable than the corresponding step leading to OCHO* across all C_60_‐based catalysts (Figure S11), with a strong pH correlation (Figure 4e–h). Higher pH environments generally lower the Gibbs free energy (ΔG) for COOH* formation, making CO production more efficient (Figure S12). Furthermore, when a specific potential is applied, the pH's influence on thermodynamic trends remains consistent. At pH = 14, the ΔG for COOH* formation decreases to 0.33 eV, which is lower than the 0.52 eV required at pH = 7.6 with U = −1.1 V_RHE_, confirming a strong pH‐dependent performance of Cu(111)‐H‐C_60_ for CO_2_RR.

### pH‐Dependent Microkinetic Volcano

Based on the simulations of COOH* adsorption energies, we constructed conventional volcano activity models to compare the catalytic activities of the COOH‐pathway (represented by G_COOH*_ and G_CO*_) and the OCHO‐pathway (G_OCHO*_ and G_HCOOH*_) (Figure S13). The result indicates that CO production is thermodynamically more favorable than HCOOH under comparable conditions. Notably, C_60_‐based catalysts appear closer to the volcano summit of the COOH‐pathway, suggesting that fine‐tuning of potential and pH can push C_60_‐based catalysts towards an optimal CO formation performance, especially under neutral and mild conditions. However, traditional volcano model is unable to capture the pH effects on catalytic behavior, as it treats the pH‐correction term in the reaction energetics as −0.059 * pH eV, which shifts in the same way as the potential shifts in the SHE scale and hard to explain the pH‐dependency in experiments under an RHE scale.^[^ ^21^ ^]^ To overcome this limitation, we developed a model that integrates both pH and electric field effects, enabling us to directly observe how pH modulates the stability and formation energies of key intermediates, thus offering deeper insights into the intrinsic catalytic properties of C_60_‐based materials. Details of the microkinetic modeling methods can be found in the Supplementary Information.

**Figure 5:**
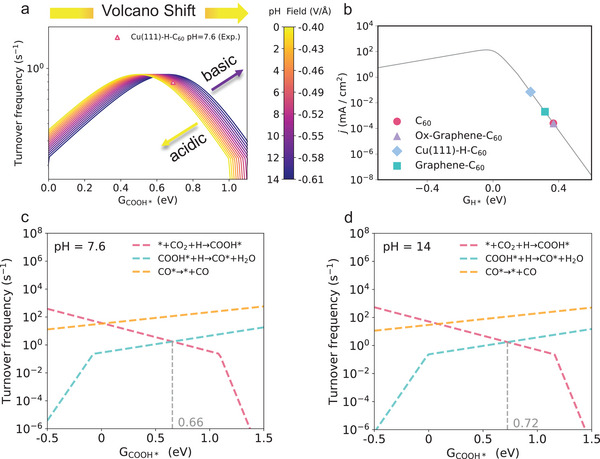
pH‐dependent microkinetic CO_2_RR volcano models at the RHE scale and rate‐determining analysis of C_60_‐based catalysts. a) Activity volcano for overall CO_2_RR turnover frequency (TOF) of C_60_‐based catalysts at −1.1 V_RHE_. b) Activity kinetic volcano for HER competition of C_60_‐based catalysts. c) Rate‐determining step (RDS) analysis of CO_2_RR activity in neutral media (pH = 7.6) and d) alkaline media (pH = 14). The vertical gray line marks the COOH* adsorption energy corresponding to the volcano summit. The pink, blue, and orange lines represent domains where CO_2_‐to‐COOH*, COOH*‐to‐CO*, and CO* desorption, respectively, serve as the rate‐limiting steps.

On basis of the insights gained from Figures 3 and 4, which examined the effects of PZCs, solvation, and electric field, Figure 5 introduces a comprehensive microkinetic model that consolidates these factors to affect the reaction mechanisms of CO_2_RR on C_60_‐based catalysts. Figure 5a displays the pH‐dependent activity volcanos, demonstrating enhanced CO_2_RR turnover frequency (TOF) in neutral and alkaline media, with a significant drop on activity observed in acidic environments due to the left‐shift of the volcano peaks. This model is based on the linear relationship between CO* and COOH* adsorption energies (Figure S14), and aligns well with our experiment observations (Figure 2a). While the double‐layer capacitance in this model is treated as constant, we acknowledge its inherent potential dependence in physical systems. Crucially, within the strong electric field applied in our study, the relative variation of capacitance becomes negligible, justifying this simplification for the present analysis. Figure 5b further extends this analysis to the HER (based on the modeling method developed in Ref. [32]), demonstrating the competitive interplay between HER with CO_2_RR. In acidic conditions, HER can prevail due to higher proton availability, but under alkaline conditions, C_60_‐based catalysts suppress HER more effectively, favoring higher CO_2_RR selectivity.

A critical aspect of our analysis lies in identifying the rate‐determining step (RDS) under varying pH conditions. As depicted in Figures 5c,d and S15, when the adsorption free energy of COOH* exceeds 0.66 eV, the initial activation of CO_2_ to form COOH* becomes the RDS in neutral media (pH = 7.6), thereby imposing a significant limitation on CO_2_RR activity. However, in alkaline media (pH = 14), the RDS shifts toward CO* formation in this potential region, driven by the effective stabilization of COOH* intermediates through reduced adsorption energies. While this transition establishes CO* formation as the kinetic bottleneck, the lowering of the activation energy for COOH* adsorption appropriately accelerates overall reaction, ultimately enhancing the overall CO production. This shift underscores a strong pH dependence, where higher pH values favor the stabilization of COOH* intermediates and enhance overall CO production. These findings align with the pH‐sensitive trends in the thermodynamic analysis shown in Figure 4, where the lowering of Gibbs free energy (ΔG) facilitates the conversion of COOH* to CO under alkaline conditions.

The comprehensive pH‐field coupled microkinetic analysis enables to study the effect of both pH and precise control over G_COOH*_ as pivotal factors influencing the catalytic performance of C_60_‐based catalysts. The study reveals that alkaline environments significantly enhance the formation and stabilization of COOH* intermediates, shifting the RDS to a step that promotes more efficient CO production. Therefore, the strategic adjustment of pH and applied potential can effectively lower energy barriers, thereby enhancing both the selectivity and efficiency of CO_2_RR. These findings make C_60_‐based catalysts highly promising candidates for sustainable CO_2_ conversion applications.

## Conclusion

In summary, C_60_ is revealed to serve as a molecular active catalytic site rather than just an electronic modulator. Traditional models overlook this complexity and fail to accurately predict the pH‐dependent performance of CO_2_RR under the experimental RHE scale. Alternatively, by integrating pH and electric field effects into a comprehensive microkinetic model, it can be concluded that C_60_’s unique structure stabilizes COOH* intermediates, particularly under alkaline conditions, enhancing CO_2_ conversion. The derived pH‐field coupled microkinetic volcano model provides a pH‐dependent framework under RHE scale, revealing a shift in activity from acidic to alkaline conditions and a transition in the RDS. This work highlights the importance of quantitative pH‐dependent modeling and provides new guidance for designing C_60_‐based catalysts. In addition, our study demonstrates that the large dipole moment change upon adsorption at curved surfaces should not be dismissed when analyzing the pH‐dependent bonding strength and electrocatalytic activity. The present work clarifies C_60_’s role in electrocatalytic CO_2_RR and exemplifies a new tool to study the catalysis under real electrochemical conditions, especially for those catalysts with curved structures.

## Author Contributions

All authors have given approval to the final version of the manuscript and declare no competing financial interest.

## Conflict of Interests

The authors declare no conflict of interest.

## Supporting information



Supporting Information

## Data Availability

All data are available upon reasonable request from the authors. Besides, the key experimental data and computational structures are available in the Digital Catalysis Platform (*DigCat*: www.digcat.org).
