# Supplementary material for: C60 Fullerene as the Active Site for CO2 Electroreduction
Source: Angew Chem Int Ed Engl. 2025 Jul 25;64(39):e202511924. doi: 10.1002/anie.202511924 (PMC12455448; doi:10.1002/anie.202511924)
Supplement: Supplementary file 1 — Supporting Information [file ANIE-64-e202511924-s001.docx]

**Supplementary Information**

**C_60_ Fullerene as the Active Site for CO_2_ Electroreduction**

Si-Wei Ying ^1,2^, Yuhang Wang ^1^, Peng Du ^2^, Qiang Wang ^3^, Changming Yue ^4^, Di Zhang ^1^, Zuo-Chang Chen ^2^, Jianwei Zheng ^2,^*, Su-Yuan Xie ^2,^*, and Hao Li ^1,^*

^1^ Advanced Institute for Materials Research (WPI-AIMR), Tohoku University, Sendai, 980-8577, Japan

^2^ State Key Laboratory of Physical Chemistry of Solid Surfaces, iChEM (Collaborative Innovation Center of Chemistry for Energy Materials), College of Chemistry and Chemical Engineering, Xiamen University, Xiamen, 361005, China

^3^ State Key Laboratory of Coal Conversion, Institute of Coal Chemistry, Chinese Academy of Sciences, Taiyuan, Shanxi 030001, China

^4^ Department of Physics, Southern University of Science and Technology, Shenzhen 518055, China

* Corresponding Authors:

[wqiang@sxicc.ac.cn](mailto:wqiang@sxicc.ac.cn) (W. Q.); [jwzheng@xmu.edu.cn](mailto:jwzheng@xmu.edu.cn) (J. W. Z.); [syxie@xmu.edu.cn](mailto:syxie@xmu.edu.cn) (S. Y. X.); [li.hao.b8@tohoku.ac.jp](mailto:li.hao.b8@tohoku.ac.jp) (H. L.)

**Supplementary Information**

[**1. Experimental methods** 4](#_Toc202719168)

[**1.1 Synthesis of Cu/SiO_2_ and C_60_-Cu/SiO_2_ catalysts** 4](#_Toc202719169)

[**1.2 Characterizations of Cu/SiO_2_ and C_60_-Cu/SiO_2_ catalysts** 4](#_Toc202719170)

[**1.3 Electrocatalytic CO_2_RR measurements** 5](#_Toc202719171)

[**1.4 Turnover frequency calculation** 6](#_Toc202719172)

[**2. Computational methods** 7](#_Toc202719173)

[**2.1 Density functional theory (DFT) calculation method** 7](#_Toc202719174)

[**2.2 Surface Pourbaix calculation** 7](#_Toc202719175)

[**2.3 Free energy calculation of CO_2_ electroreduction** 8](#_Toc202719176)

[**2.4 Electric fields and pH-dependent modeling at the RHE-scale** 9](#_Toc202719177)

[**2.5 Determining the potential of zero charge (PZC)** 10](#_Toc202719178)

[**2.6 Charge density difference calculation** 11](#_Toc202719179)

[**2.7 Microkinetic modeling** 11](#_Toc202719180)

[**3. Experimental summary of catalysts for CO_2_RR** 13](#_Toc202719181)

[**4. Charicterization of catalysts** 17](#_Toc202719182)

[**5. Faradaic efficiency for CO_2_RR of C_60_ catalyst** 18](#_Toc202719183)

[**6. Surface Pourbaix diagram** 19](#_Toc202719184)

[**7. Dipole moment (μ) and polarizability (α) of COOH* and OCHO* on Cu(111) surface** 20](#_Toc202719185)

[**8. Change in relative Gibbs free energy of COOH* on planar graphene surface** 21](#_Toc202719186)

[**9. Most stable adsorption configurations** 22](#_Toc202719187)

[**10. Field effect on C_70_ surface** 23](#_Toc202719188)

[**11. Bader charge analysis** 24](#_Toc202719189)

[**12. Band structures and band gaps** 25](#_Toc202719190)

[**13. Comparison of implicit and explicit PZCs** 26](#_Toc202719191)

[**14. Free energy diagrams for COOH* and OCHO* formation with U = 0 V_RHE_** 27](#_Toc202719192)

[**15. Free energy diagrams for COOH-Path with U = 0 V_RHE_** 28](#_Toc202719193)

[**17. Scaling relationships of different adsorbates** 30](#_Toc202719194)

[**18. RDS analysis** 31](#_Toc202719195)

[**19. Thermodynamic corrections** 32](#_Toc202719196)

[References 33](#_Toc202719197)

**1. Experimental methods**

**1.1 Synthesis of Cu/SiO_2_ and C_60_-Cu/SiO_2_ catalysts**

The C_60_-Cu/SiO_2_ catalyst was synthesized using a urea-assisted gelation procedure. The content of C_60_-Cu/SiO_2_ with C = 0.03, Cu = 0.2, and SiO_2_ = 0.77 in wt%. 6.8 g of Cu(NO_3_)_2_⋅3H_2_O was dissolved in 100 ml of suitably diluted 28 wt% aqueous ammonia in a round bottom flask. A certain amount of C_60_ (1.02 g) was introduced into the obtained Cu ammonium solution and ultrasonic stirring the mixture till the formation of the suspension. Subsequently, 3.0 g of urea was added prior to slowly dropping 40 wt% Ludox AS-40 colloidal silica with a burette under mechanical stirring. Then the mixture was vigorously stirred and aged at 90 °C in an oil bath for 4 h. The obtained light blue precipitate was separated by ﬁltration, washed with deionized water and ethanol, dried under vacuum overnight at 120 °C, and then calcined at 350 °C in the air for 4 h. C_60_ is stable under this condition. Similar procedure for the synthesis of pristine Cu/SiO_2_ excluding the introduction of C_60_. All the catalysts were reduced at 300 °C for 4 h in 5% H_2_–95% N_2_ atmosphere and then cooled to room temperature prior to characterizations.

**1.2 Characterizations of Cu/SiO_2_ and C_60_-Cu/SiO_2_ catalysts**

SEM, TEM images, and XRD patterns of catalysts were conducted. SEM images were observed on a Phenom Pro microscope operating at an acceleration voltage of 10 kV. TEM images were observed on a Tecnai F30 apparatus operating at an acceleration voltage of 300 kV. Spherical aberration corrected transmission electron microscopy (AC-TEM) and electron energy loss spectroscopy (EELS) analysis were performed on a JEM-ARM200F scanning transmission electron microscope operated with a probe spherical aberration (Cs) corrector working at 80 kV. The instrument was equipped with a Gatan Quantum 965 image filter system. The catalyst samples were processed in a sealed flask filled with N_2_. After reduction and cool down, ethanol was injected into the flask and ultrasonically dispersed at room temperature for 30 min. A drop of this solution was trickled on the copper grid for TEM image measurements. XRD patterns for the catalyst samples were obtained on a PANAlytical X’pert Pro Super X-ray diffractometer using Cu K( radiation (λ = 0.15418 nm) with a scanning angle (2θ) range of 10° to 90°, a tube voltage of 40 kV, and a current of 30 mA. The full-width-at-half-maximum (FWHM) of Cu(111) diffraction was used to calculate the Cu crystallite size using the classic Scherrer equation. The crystalline phases on the catalysts were identified by matching the collected diffraction pattern with the reference patterns included in the JCPDS database.

**1.3 Electrocatalytic CO_2_RR measurements**

5 mg of catalyst and 2 mg of Ketjen-black were dispersed into isopropanol alcohol (940 μl) and Nafion solution (60 μl), followed by mixing with the assistance of ultrasonication for 30 min to achieve a homogeneous ink. 100 μl of the catalyst ink was pipetted onto a carbon paper electrode (1×1 cm^2^) and dried at room temperature. The electrochemical measurements were performed in a H-type cell with two-compartments separated by anion exchange membrane (Nafion-117) by CHI 660E electrochemical analyzer (CH Instruments, Inc.) at room temperature. One compartment contained 70 ml electrolyte (0.1 M KHCO_3_ aqueous solution) and Pt foil as counter electrodes, another with the same electrolyte, Ag/AgCl electrode in saturated KCl solution as reference electrodes and working electrode. During the electrochemical measurements, the electrolyte solution was purged with CO_2_ for 30 min to achieve the CO_2_-saturated solution (pH = 7.6). All recorded potentials were recalibrated to correspond with the reversible hydrogen electrode as per the following equation:

$E(V\text{ }\text{vs}\text{ }RHE)=E(V\text{ }\text{vs}\text{ }Ag/AgCl)+0.197(\text{ }V)+0.059\times pH$ (1)

CO_2_ gas was delivered at an average rate of 30 ml min^–1^ and routed into the gas sampling loop of a gas chromatograph. The gas phase composition was analyzed by GC every 15 min. The separated gas products were analyzed by a thermal conductivity detector (for H_2_) and a flame ionization detector (for CO, CH_4_, and C_2_H_4_). The Faraday efficiency of gas products was calculated by the following equation:

$\mathrm{FE}_{g}=\frac{n\times96485 \left( c/\mathrm{mol} \right)\times V \left( \mathrm{mL}/\min\right)\times{10}^{-6}({m^{3}}/\mathrm{mL})\times v(vol \%)\times1.013\times{10}^{5}(N/{m^{2}})}{8.314\left( {N\cdot m}/{mol\cdot K} \right)\times298.15 (K)\times I_{\mathrm{total}}(C/s)\times60(s/\min)}$ (2)

ν (vol %) = Volume concentration of CO in the exhaust gas from the H-cell (GC data).

V (ml/min) = Gas flow rate measured by a flow meter at the exit of the cell at room temperature and under ambient pressure.

I_total_ (C/s) = Steady-state cell current.

**1.4 Turnover frequency calculation**

The TOF value was calculated as follows:

$TOF=\frac{J_{\mathrm{CO}}/nF}{m_{\text{catalyst}}\times\omega/M_{\text{active}}}\times3600$ (3)

where $J_{CO}$ is the partial current for CO $(A),n$ is the number of electrons transferred for product formation (here $n=2$ ), $F$ is the Faraday constant (96485$\left. {C mol}^{-1} \right)$, $m_{\text{catalyst}}$ is the mass of catalyst on the electrode $(g)$, $\omega$ is the active component loading in the catalyst (wt.%), and $M_{\text{active}}$ is atomic mass of the active component.

**2. Computational methods**

**2.1 Density functional theory (DFT) calculation method**

In this work, all theoretical data were executed employing the spin-polarization density functional theory (DFT) method incorporating van der Waals corrections, as implemented in the Vienna *ab initio* simulation package (VASP)^1^. VASP 6.3.2 version was used here. The exchange-correlation functional was approximated by the rivesed Perdew-Burke-Ernzerhof (RPBE) functional within the generalized gradient approximation (GGA) method^2^. The electronic wave functions were expanded within a plane-wave basis set with a cutoff energy of 500 eV. For the surface models, a vacuum layer of 15 Å was used to simulate the catalyst surface to avoid interactions between periodic images. The van der Waals correction was considered as the zero-damping variant of DFT-D3. During optimization of structure which containing metal, the bottom two layers of the substrate were fixed. The Brillouin zone integration was sampled by 1×1×1 Γ-centered k-point mesh grid for the surface model. Convergence criteria for energies and forces were set at 10^–5^ eV, and all structural relaxation persisted until forces on atoms were less than 0.05 eV/Å.

**2.2 Surface Pourbaix calculation**

Based on the examination of potential surface states of exposed Cu(111) surfaces, the free energies shown in the surface Pourbaix diagrams ($G_{SP}$) were calculated using the method outlined in Ref.^3-5^,

$G_{SP}=G_{\text{pristine}}+mG_{H_{2}O}-G_{\text{tot}}-(2m-n)\left( \frac{1}{2}G_{H_{2}}-U_{SHE}-2.303k_{B}T*pH \right)$ (4)

where $G_{\text{pristine}}$ represents the total free energy of a pristine surface, where $G_{H_{2}O}$ is the free energy of H_2_O, $G_{\text{tot}}$ denotes the total free energy of the surface with adsorbate or non-metal vacancies, $G_{H_{2}}$ is the total energy of molecular hydrogen (H_2_).The variable $m$ and $n$ signify the numbers of oxygen and hydrogen atoms adsorbed onto a stoichiometric surface, respectively. $k_{B}$ is the Boltzmann constant, and $T$ is the temperature.

**2.3 Free energy calculation of CO_2_ electroreduction**

The adsorption free energy of reaction intermediates during CO_2_ reduction reaction (CO_2_RR) was defined as:

$\Delta G\left( \mathrm{COOH}{}^{*} \right)=G\left( \mathrm{COOH}{}^{*} \right)-G\left( {}^{*} \right)-G(CO_{2})-\frac{1}{2}G_{H_{2}}$ (5)

$\Delta G\left( \mathrm{CO}{}^{*} \right)=G\left( \mathrm{CO}{}^{*} \right)-G\left( \mathrm{COOH}{}^{*} \right)-G(H_{2}O)-\frac{1}{2}G_{H_{2}}$ (6)

$\Delta G\left( \mathrm{des}{}^{*} \right)=G\left( \mathrm{CO} \right)-G\left( \mathrm{CO}{}^{*} \right)$ (7)

$\Delta G\left( \mathrm{OCHO}{}^{*} \right)=G\left( \mathrm{OCHO}{}^{*} \right)-G\left( {}^{*} \right)-G(CO_{2})-\frac{1}{2}G_{H_{2}}$ (8)

$\Delta G\left( \mathrm{HCOOH} \right)=G\left( \mathrm{HCOOH}{}^{*} \right)-G\left( \mathrm{OCHO}{}^{*} \right)-\frac{1}{2}G_{H_{2}}$ (9)

where $G\left( \mathrm{COOH}{}^{*} \right)$ and $G\left( {}^{*} \right)$ mean the free energy of $\mathrm{COOH}{}^{*}$ species adsorbed on the surface and slab surface $\left( {}^{*} \right)$, respectively. The calculation of $G(CO_{2})$，$G_{H_{2}}$ , and $G(H_{2}O)$ refer to the energies of $\mathrm{CO}_{2}$, $H_{2}$, and $H_{2}O$, respectively. The free energy of each step for $\mathrm{CO}_{2}\mathrm{RR}$ was defined by employing previous methods by Nørskov and co-workers^6^:

$\Delta G=\Delta E+\Delta E_{\mathrm{ZPE}}+\int C_{p}dT-T\Delta S +E_{solv}$ (10)

where $\Delta E$ means the difference in DFT electronic energy, $\Delta E_{\mathrm{ZPE}}$, $\int C_{p}\mathrm{dT}$ and $\Delta S$ refer to the zero point energy, heat capacities, and entropy corrections, and $T$ is the absolute temperature, set to $298.15\text{ }K$. The zero point energy and entropic contribution $(T\cdot S)$ were obtained with the harmonic approximation. $E_{solv}$ is the solvation corrections described in AIMD simulations of explicit water for COOH* and OCHO* intermediates and Ref.^7^ for CO*, with Δ$E_{solv}$(COOH) = -0.57 eV, Δ$E_{solv}$(CO) = -0.25 eV, Δ$E_{solv}$(OCHO) = -0.20 eV.

**2.4 Electric fields and pH-dependent modeling at the RHE-scale**

To understand the response of adsorbates under different fields, electric fields with ranging from -0.6 to 0.6 V/Å were applied using a saw-tooth potential. The adsorbates were relaxed to reach a force convergence threshold of 0.05 eV/Å during each field application. The lowest energy configurations identified under these conditions were used to estimate the adsorbate energies. A second-order polynomial was fitted to the energy calculations across the field range for each adsorbate. This allows the extraction of intronsic dipole moment (μ) and polarizability (α) by Equation 11,

$G_{ads}=G_{ads}^{PZC}+\mu\vec{E}-\frac{\alpha}{2}\vec{E}^{2}$ (11)

where $G_{ads}$ is the adsorption energy of adsorbate under electric field and $\text{G}_{\text{ads}}^{\text{PZC}}$ is the adsorption energy of adsorbate at PZC. Since we used fixed PZC and C_H_ values, the adsorption energy dependence on the SHE potential is distinct from that of Ref.^8^, whose approach uses implicit solvent methods and allows for these parameters to vary with adsorption. This has raised questions about the accuracy of their predictions, as several studies has noted^9, 10^.

A parallel-plate capacitor model was used to link the electric fields with the standard hydrogen electrode (SHE) and reversible hydrogen electrode (RHE) potentials, accounting for the potential and pH effects. The model is described by Equation 12,

$\vec{E}=\frac{\sigma}{\varepsilon\varepsilon_{0}}=\frac{C_{H}\left( U_{SHE}-U_{PZC} \right)}{\varepsilon\varepsilon_{0}}$ (12)

In this equation, *σ* is the charge density, $\varepsilon_{0}$ is the vacuum permittivity (8.85 × 10^−12^ F m^−1^), $\varepsilon$ is the dielectric constant (unitless). As demonstrated by Fumagalli et al., the dielectric constant of water near a surface is 2^11^. *C_H_* is the Helmholtz capacitance (μF cm^−2^), *U_SHE_* refers to the potential *vs*. SHE, and *U_PZC_* refers to the potential at the potentials of zero charge (PZCs) *vs*. SHE, respectively.

The computational hydrogen electrode (CHE) model was used to adjust the adsorption energies for RHE dependence using equation 10, where *n* refers to the number of electrons, *e* refers to the charge of an electron, and *U_RHE_* refers to the potential *vs.* RHE.

$G_{ads}=G_{ads,U_{RHE=0}}-neU_{RHE}$ (13)

Ultimately, the free energy of adsorbate at the given *U_RHE_* and *U_SHE_* is shown by Equation 14:

$G_{ads}=G_{ads}^{PZC}+\mu\frac{C_{H}(U_{SHE}-U_{PZC})}{\varepsilon\varepsilon_{0}}-\frac{\alpha}{2}\left( \frac{C_{H}(U_{SHE}-U_{PZC})}{\varepsilon\varepsilon_{0}} \right)^{2}-neU_{RHE}$ (14)

**2.5 Determining the potential of zero charge (PZC)**

To predict the values of PZCs, previous studies have relied on implicit solvation model (such as VASPsol^12, 13^) or DFT calculations with a static layer of water molecules. However, Schnur and Groß^14^ showed that these values are highly depend on the initial orientation of the water molecules first spotted in the simulation. AIMD simulations of metal-water interfaces have emerged as a more accurate method to estimate PZCs in order to address this issue^14-17^.

In this work, we analyzed the computational PZCs for all C_60_-based catalysts using both implicit solvation and explicit solvation models, namely VASPsol and AIMD. For implicit models, VASPsol parameters were set to their default values, which include a bulk dielectric constant *ε_k_* = 78.4, width of dielectric cavity σ = 0.6, cutoff charge density ρ_cut_ = 0.0025 Å^−3^, and a surface tension parameter of 0.525 meV/Å^2^. AIMD simulations were performed using VASP with the RPBE exchange-correlation functional and D3 dispersion correction scheme^18^, which is known for its precision in describing metal−water interfaces. The electronic wave functions were expanded within a plane-wave basis set with a cutoff energy of 500 eV. Convergence criteria for energies and forces were set at 10^–5^ eV, and all structural relaxation persisted until forces on atoms were less than 0.05 eV/Å. *Γ*-point AIMD simulations were performed with a 2 fs time step, and a Nosé thermostat was maintained at 298 K. The catalyst−water interfaces for all C_60_-based catalysts comprised a water layer of at least 10 Å, corresponding to a water density of 1 g/cm^3^.

Trasatti *et al.*^19^ showed that U_PZC_ could be directly derived from the work function of a material in ion-free water *ϕ* using Equation 15:

$\phi=eU_{PZC}+e\phi_{SHE}$ (15)

where *ϕ_SHE_* is the absolute potential energy of the SHE. The value of *ϕ_SHE_* can vary depending on the experiment conducted (ranging from 4.3 to 4.8 eV). Note that the International Union of Pure and Applied Chemistry (IUPAC) approved value of 4.44 eV was employed in this work.

**2.6 Charge density difference calculation**

The charge density differences $(\Delta\rho$) induced by COOH* were plotted using the following equation:

$\Delta\rho=\rho_{\text{tot}}-\rho_{\text{pristine}}-\rho_{COOH}$ (16)

where $\rho_{\text{tot}}$ is the charge density of the surface with COOH adsorbed onto the active site, $\rho_{\text{pristine}}$ is the charge density of the clean surface, and $\rho_{COOH}$ is the charge density of the COOH molecule.

**2.7 Microkinetic modeling**

The microkinetic modeling of the CO_2_RR volcano was based on the strategy outlined by Hansen *et al.*^20^ and Kelly *et al.*^21^. Rates for intermediate steps were calculated using Equation 17,

$rate=k_{f}\prod\theta_{reac}-k_{b}\prod\theta_{prod}$ (17)

where $\text{θ}_{\text{reac}}$ and $\text{θ}_{\text{prod}}$ are the coverages of reactants and products, respectively. $k_{f}$ and $k_{b}$ are the rate constant of forwards and backwards reaction, which were calculated as the function of reaction perfector A (s^-1^), activation free energy *G_a_*, Boltzmann constant $k_{B}$, and reaction temperature *T*:

$k=Ae^{-\frac{G_{a}}{k_{B}T}}$ (18)

The intermediate for CO formation considered in the modeling are shown in Reactions (19-21):

${CO}_{2}\left( g \right) + * + H^{+} + e^{-}\to\mathrm{COOH}{}^{*}$ (19)

$\mathrm{COOH}{}^{*} +H^{+} + e^{-} \to\mathrm{CO}{}^{*}+H_{2}O$ (20)

$\mathrm{CO}{}^{*}\to CO + *$ (21)

The intermediate for HCOOH formation considered in the modeling are shown in Reactions (22-23):

${CO}_{2}\left( g \right) + * + H^{+} + e^{-} \to\mathrm{OCHO}{}^{*}$ (22)

$\mathrm{OCHO}{}^{*} + H^{+} + e^{-} \to* + HCOOH$ (23)

Reactions (19-21) involve proton-electron transfer steps, where the energy of the proton-electron pair is represented by the energy of half of a H_2_ molecule according to the CHE method^6^. For proton transfers, we used an intrinsic barrier described in Ref.^20^. Perfectors for all of the proton-electron transfer steps were set as 1×10^9^ s^−1^ to account for solvent reorganization^21^.

**3.** **Experimental summary of catalysts for CO_2_RR**

**Table S1.** Summary for experimentally reported typical catalysts for CO_2_RR.

| Formula | Type | Electrolytes | Faradaic efficiency  (% @ U V_RHE_) | Reference |
| --- | --- | --- | --- | --- |
| CuPc | M-N-C | 0.1 M KHCO_3_ | 31.25%@-0.5 | ^22^ |
| Cu-N-C | M-N-C | 0.5 M KHCO_3_ | 19%@-0.9 | ^23^ |
| CuN_3_O/C SAC | M-N-C | 0.1 M KHCO_3_ | 96%@-0.8 | ^24^ |
| CuCO_3_/C SAC | M-N-C | 0.1 M KHCO_3_ | 20%@-0.5 | ^24^ |
| Cu-GS-800 | M-N-C | 0.1 M KHCO_3_ | 59%@-0.8 | ^25^ |
| Cu-GS-900 | M-N-C | 0.1 M KHCO_3_ | 34%@-0.8 | ^25^ |
| Cu-GS-1000 | M-N-C | 0.1 M KHCO_3_ | 12%@-0.8 | ^25^ |
| SF-Cu/CA | M-N-C | 0.1 M KHCO_3_ | 58.43%@-1.26 | ^26^ |
| SF-Cu/CA-1 | M-N-C | 0.1 M KHCO_3_ | 81.03%@-1.26 | ^26^ |
| CoPc-Cu-O | M-N-C | 0.2 M KHCO_3_ | 85%@-0.74 | ^27^ |
| CoPc-Cu-NH | M-N-C | 0.2 M KHCO_3_ | 72%@-0.74 | ^27^ |
| NiPc-Cu-O | M-N-C | 0.2 M KHCO_3_ | 56%@-0.74 | ^27^ |
| NiPc-Cu-NH | M-N-C | 0.2 M KHCO_3_ | 43%@-0.74 | ^27^ |
| CuNi_AC_@Ni/Cu-N-C | M-N-C | 0.1 M KHCO_3_ | 98.2%@-0.7 | ^28^ |
| Cu-S-Ni/SNC | M-N-C | 0.1 M KHCO_3_ | 98.1%@-0.65 | ^29^ |
| CuNi/NC | M-N-C | 0.1 M KHCO_3_ | 73%@-0.65 | ^29^ |
| Cu/SNC | M-N-C | 0.1 M KHCO_3_ | 58%@-0.65 | ^29^ |
| CuZn-DAS/NC | M-N-C | 1 M KOH | 98.4%@-0.6 | ^30^ |
| Cu-SAS/NC | M-N-C | 1 M KOH | 36.4%@-0.6 | ^30^ |
| Cu/Ni-NC | M-N-C | 0.5 M KHCO_3_ | 99.1%@-1.1 | ^31^ |
| TeN_2_-CuN_3_ | M-N-C | 0.1 M KHCO_3_ | 98%@-0.65 | ^32^ |
| CuN_4_ | M-N-C | 0.1 M KHCO_3_ | 60%@-0.85 | ^32^ |
| Ni/Cu_0.38_-CTF | M-N-C | 0.1 M KHCO_3_ | 99.1%@-1.1 | ^33^ |
| Cu-CTF | M-N-C | 0.1 M KHCO_3_ | 30%@-0.8 | ^33^ |
| Cu_2_/NC | M-N-C | 0.5 M KHCO_3_ | 33%@-0.63 | ^34^ |
| Cu_1_/NC | M-N-C | 0.5 M KHCO_3_ | 57%@-0.63 | ^34^ |
| Cu-N-C | M-N-C | 0.5 M KHCO_3_ | 70%@-0.6 | ^35^ |
| Cu-N-C | M-N-C | 0.1 M KHCO_3_ | 14%@-0.95 | ^36^ |
| NiCu-NC | M-N-C | 0.1 M KHCO_3_ | 98%@-1.17 | ^37^ |
| Cu-NC | M-N-C | 0.1 M KHCO_3_ | 53%@-0.67 | ^37^ |
| Cu-ZNC | M-N-C | 0.1 M KHCO_3_ | 80%@-0.7 | ^38^ |
| Ni/Cu-N-C | M-N-C | 0.5 M KHCO_3_ | 97.7%@-0.6 | ^39^ |
| Cu-N-C | M-N-C | 0.5 M KHCO_3_ | 42.6%@-0.6 | ^39^ |
| CuNi-DSA/CNFs | M-N-C | 0.1 M KHCO_3_ | 99.6%@-0.98 | ^40^ |
| Cu-SA/CNFs | M-N-C | 0.1 M KHCO_3_ | 20.6%@-1.08 | ^40^ |
| Cu-N-C | M-N-C | 0.1 M KHCO_3_ | 30%@-0.76 | ^41^ |
| Cu/Ni-NC | Metal/Alloy | 0.5 M KHCO_3_ | 93.2%@-0.7 | ^42^ |
| Cu-NC | Metal/Alloy | 0.5 M KHCO_3_ | 55%@-0.4 | ^42^ |
| CuAg/CeO_2_-6 | Metal/Alloy | 0.1 M KHCO_3_ | 84%@-1.1 | ^43^ |
| Cu/CeO_2_ | Metal/Alloy | 0.1 M KHCO_3_ | 42%@-1.1 | ^43^ |
| Pd_1_Cu_1_ | Metal/Alloy | 0.1 M KHCO_3_ | 97%@-0.8 | ^44^ |
| Pd_1_Cu_1_ | Metal/Alloy | 1 M KOH | 96%@-0.88 | ^44^ |
| Sn-CuO-7.5 | Metal/Alloy | 1 M KOH | 95.5%@-0.7 | ^45^ |
| Ag_83_Cu_17_ | Metal/Alloy | 0.1 M KHCO_3_ | 74%@-1.1 | ^46^ |
| Ag_43_Cu_57_ | Metal/Alloy | 0.1 M KHCO_3_ | 15%@-0.9 | ^46^ |
| CuIn NWs | Metal/Alloy | 0.1 M KHCO_3_ | 86%@-1.0 | ^47^ |
| Cu_90_In_10_/C | Metal/Alloy | 0.1 M KHCO_3_ | 85%@-0.75 | ^48^ |
| Cu/C | Metal/Alloy | 0.1 M KHCO_3_ | 3.1%@-0.75 | ^48^ |
| Sn/Cu cones | Metal/Alloy | 0.1 M KHCO_3_ | 82.7%@-0.6 | ^49^ |
| Sn/Cu foils | Metal/Alloy | 0.1 M KHCO_3_ | 41.3%@-0.8 | ^49^ |
| Sn/Cu rods | Metal/Alloy | 0.1 M KHCO_3_ | 59.7%@-0.8 | ^49^ |
| Cu cones | Metal/Alloy | 0.1 M KHCO_3_ | 48.8%@-0.7 | ^49^ |
| Cu foils | Metal/Alloy | 0.1 M KHCO_3_ | 11.4%@-0.7 | ^49^ |
| Cu rods | Metal/Alloy | 0.1 M KHCO_3_ | 38.7%@-0.7 | ^49^ |
| Sn/Cu_2_O NSs | Metal/Alloy | 0.1 M KHCO_3_ | 87.9%@-1.3 | ^50^ |
| Au_75_Cu_25_ | Metal/Alloy | 0.1 M KHCO_3_ | 64.4%@-0.7 | ^51^ |
| Au_50_Cu_50_ | Metal/Alloy | 0.1 M KHCO_3_ | 58.8%@-0.7 | ^51^ |
| Au_25_Cu_75_ | Metal/Alloy | 0.1 M KHCO_3_ | 28.1%@-0.8 | ^51^ |
| Cu | Metal/Alloy | 0.1 M KHCO_3_ | 17.3%@-0.6 | ^51^ |
| Cu_2_Cd/Cd/Cu | Metal/Alloy | 0.1 M KHCO_3_ | 84%@-1.0 | ^52^ |
| Cu_2_In MAs | Metal/Alloy | 0.1 M KHCO_3_ | 94.5%@-0.6 | ^53^ |
| Cu_1_In MAs | Metal/Alloy | 0.1 M KHCO_3_ | 50.94%@-0.6 | ^53^ |
| Cu_3_In MAs | Metal/Alloy | 0.1 M KHCO_3_ | 75.18%@-0.7 | ^53^ |
| Cu_4_In MAs | Metal/Alloy | 0.1 M KHCO_3_ | 67.71%@-0.6 | ^53^ |
| Cu MAs | Metal/Alloy | 0.1 M KHCO_3_ | 5%@-0.9 | ^53^ |
| np-Ag/Sn-Cu | Metal/Alloy | 1 M KOH | 99%@-0.6 | ^54^ |
| np-Ag-Cu | Metal/Alloy | 1 M KOH | 70%@-0.5 | ^54^ |
| np-Sn-Cu | Metal/Alloy | 1 M KOH | 81%@-0.6 | ^54^ |
| np-Cu | Metal/Alloy | 1 M KOH | 48%@-0.6 | ^54^ |
| Cu_92_Sb_5_Pd_3_ | Metal/Alloy | 0.5 M KHCO_3_ | 100%@-0.93 | ^55^ |
| Cu_97_Pd_3_ | Metal/Alloy | 0.5 M KHCO_3_ | 90%@-0.93 | ^55^ |
| Cu_95_Sb_5_ | Metal/Alloy | 0.5 M KHCO_3_ | 85%@-0.93 | ^55^ |
| Cu | Metal/Alloy | 0.1 M KHCO_3_ | 41%@-0.7 | ^38^ |
| InZnCu/CF | Metal/Alloy | 0.1 M KHCO_3_ | 93.7%@-0.7 | ^56^ |
| Ag NPs/CuO MNSs | Metal/Alloy | 0.1 M KHCO_3_ | 97.8%@-0.7 | ^57^ |
| CuO | Metal/Alloy | 0.1 M KHCO_3_ | 31.2%@-0.7 | ^57^ |
| Au_2_-Cu_8_ | Metal/Alloy | 0.1 M KHCO_3_ | 94%@-0.8 | ^58^ |
| Au_1_-Cu_9_ | Metal/Alloy | 0.1 M KHCO_3_ | 69%@-0.8 | ^58^ |
| Au_3_-Cu_7_ | Metal/Alloy | 0.1 M KHCO_3_ | 72%@-0.8 | ^58^ |
| CuIn20 | Metal/Alloy | 0.1 M KHCO_3_ | 93%@-0.6 | ^59^ |
| Cu NWs | Metal/Alloy | 0.1 M KHCO_3_ | 47%@-0.6 | ^59^ |
| Cu/Ni(OH)_2_ | Metal X-ides | 0.5 M NaHCO_3_ | 92%@-0.5 | ^60^ |
| Cu_3_SbS_4_ | Metal X-ides | 0.5 M KHCO_3_ | 60%@-1.0 | ^61^ |
| CuO | Metal X-ides | 1 M KOH | 30%@-0.8 | ^45^ |
| Cu_0.14_Zn_0.86_O | Metal X-ides | 0.1 M KHCO_3_ | 14%@-0.93 | ^62^ |
| Cu_0.43_Zn_0.57_O | Metal X-ides | 0.1 M KHCO_3_ | 45%@-0.95 | ^62^ |
| Cu_0.75_Zn_0.25_O | Metal X-ides | 0.1 M KHCO_3_ | 45%@-0.95 | ^62^ |
| CuO | Metal X-ides | 0.1 M KHCO_3_ | 59%@-0.95 | ^62^ |
| S0-Cu_2_O-70 | Metal X-ides | 0.1 M KHCO_3_ | 40%@-0.8 | ^63^ |
| Cu_1_Zn_9_-Ni | Metal X-ides | 1 M KOH | 80.5%@-0.8 | ^64^ |
| Cu_1_Zn_9_ | Metal X-ides | 1 M KOH | 61%@-0.8 | ^64^ |

**4. Charicterization of catalysts**

**Figure S1.** TEM images of (a) Cu/SiO_2_ and (b) C_60_-Cu/SiO_2_ catalysts and their morphologies. (c) The particle distributions of C_60_-Cu/SiO_2_ catalyst. (d) XRD patterns of Cu/SiO_2_ and C_60_-Cu/SiO_2_ catalysts. (e) AC-TEM image a selective Cu nanoparticle. (f) Line EELS profiles in the corresponding line in (e).

**5. Faradaic efficiency for CO_2_RR of C_60_ catalyst**


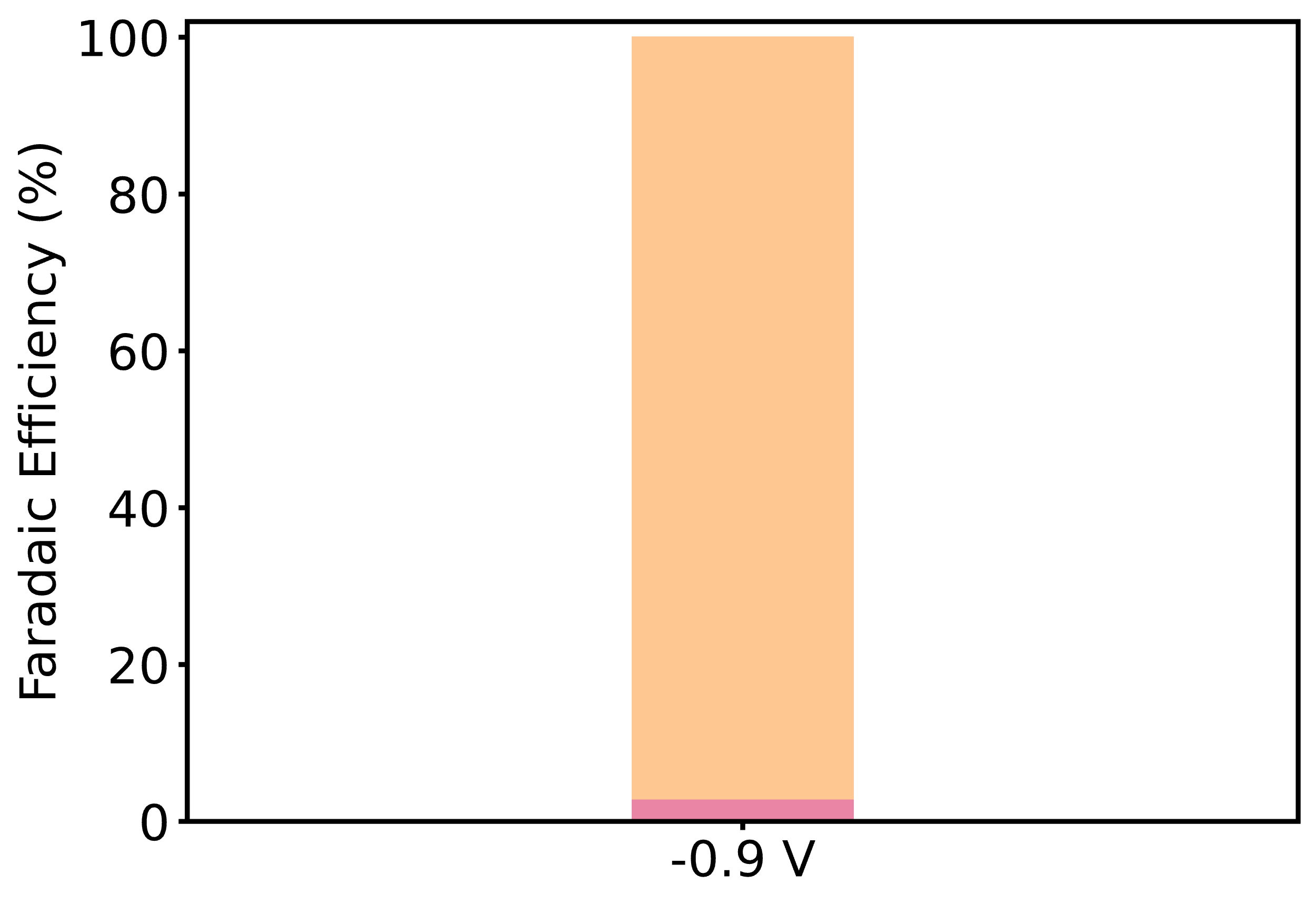


**Figure S2.** Faradaic efficiency (FE) for electrochemical CO_2_RR to CO over C_60_ catalyst.

**6. Surface Pourbaix diagram**

**Figure S3.** (a) 1D and (b) 2D surface Pourbaix diagrams of Cu(111)-H-C_60_.

**7. Dipole moment (μ) and polarizability (α) of COOH* and OCHO* on Cu(111) surface**

**Figure S4.** (a) Electric field effects on COOH* and OCHO* intermediates with the fitted values of μ (eÅ) and α (e^2^ V^−1^) on Cu(111) surface. (b) The most stable adsorption configuration of COOH* and OCHO* intermediates on Cu(111) surface.

**8. Change in relative Gibbs free energy of COOH* on planar graphene surface**

**Figure S5.** (a) Gibbs free energy change of COOH* under an applied electric field at planar graphene. (b) The most stable adsorption configuration of COOH* on planar graphene.

**9. Most stable adsorption configurations**

**Figure S6.** Most stable adsorption configurations of COOH*, OCHO*, CO*, and HCOOH* on C_60_-based catalysts. Light gray, red, brown, and pink spheres represent C, O, Cu, and H, respectively.

**10. Field effect on C_70_ surface**

**Figure S7.** (a) The structure of C_70_ fullerene. Potential active site with high curvature (b) and low curvature (c) in C_70_. (d) Relative energy difference between Site A and Site B. Electric field effects on the adsorption energies of CO_2_RR adsorbates, with fitted values for μ (dipole moment, еÅ) and α (polarizability, e^2^ V^-1^) for (e) C_70_ site A and (f) C_70_ site B. Charge difference isosurfaces value: 0.0015. Atom colors: Gray (C), red (O), and white(H).

**11. Bader charge analysis**

**Figure S8.** The Bader charge difference values of bonded C (C_60_)-C (_COOH_) under electric field.

**12. Band structures and band gaps**

**Figure S9.** Band structure analysis of (a) C_60_, (b) Graphene-C_60_, and (c) Ox-Graphene-C_60_. The band gaps of C_60_ and Graphene-C_60_ are 1.60 eV and 0.25 eV, respectively, whereas Ox-Graphene-C_60_ is in the metallic state.

**13. Comparison of implicit and explicit PZCs**

**
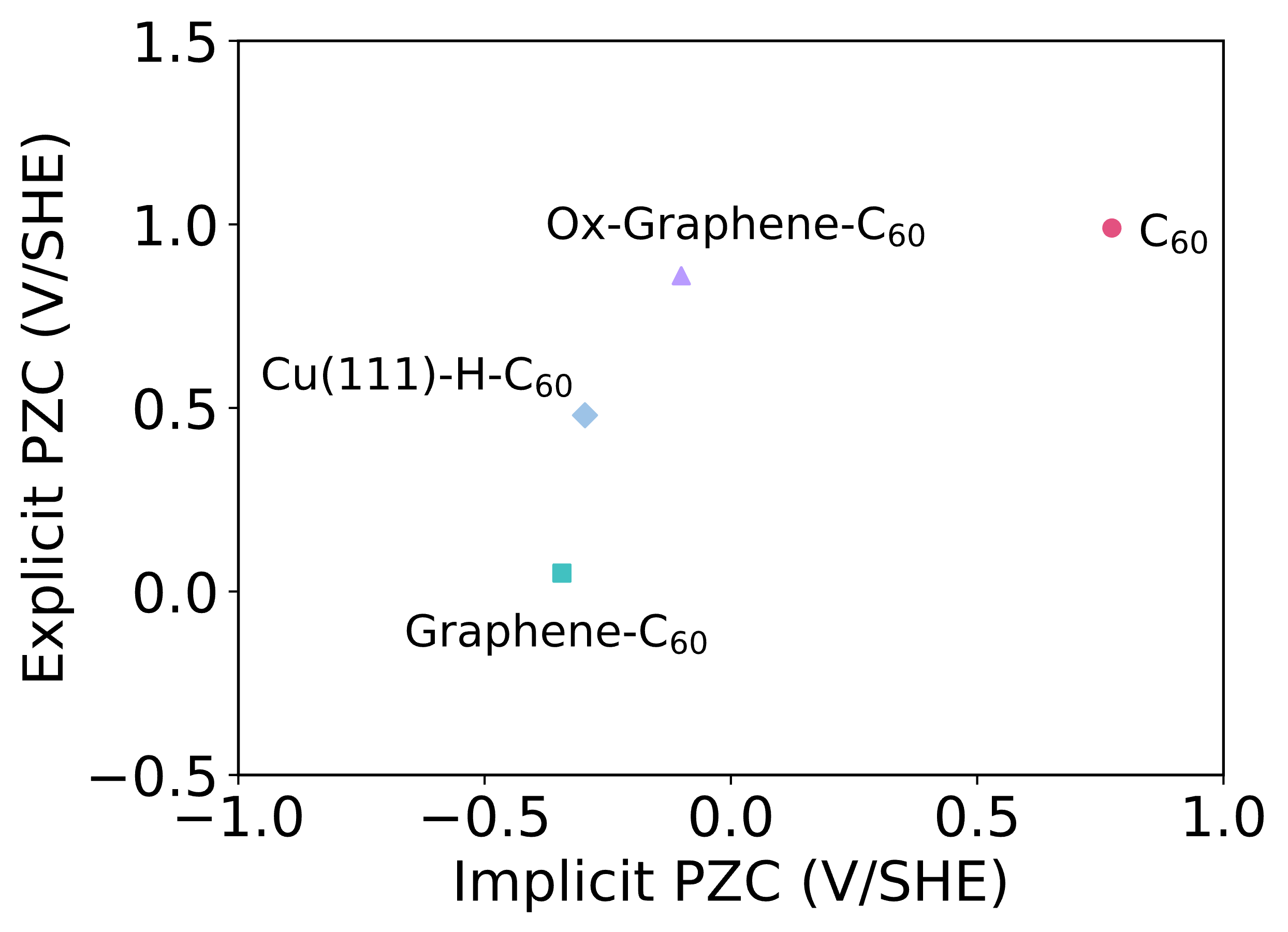
**

**Figure S10.** Comparison between the PZCs calculated from implicit and explicit solvation models.

|  | $\boldsymbol{\phi}$ **(eV )** | **PZC_implicit_ (V/SHE)** | **PZC_explicit_ (V/SHE)** | |
| --- | --- | --- | --- | --- |
| C_60_ | 5.43 | +0.77 | | +0.99 |
| Ox-Graphene-C_60_ | 5.30 | −0.10 | | +0.86 |
| Cu(111)-H-C_60_ | 4.92 | −0.30 | | +0.48 |
| Graphene-C_60_ | 4.49 | -0.34 | | +0.05 |

**Table S2**. The PZC values of implicit and explicit models.

**14. Free energy diagrams for COOH* and OCHO* formation with U = 0 V_RHE_**

**
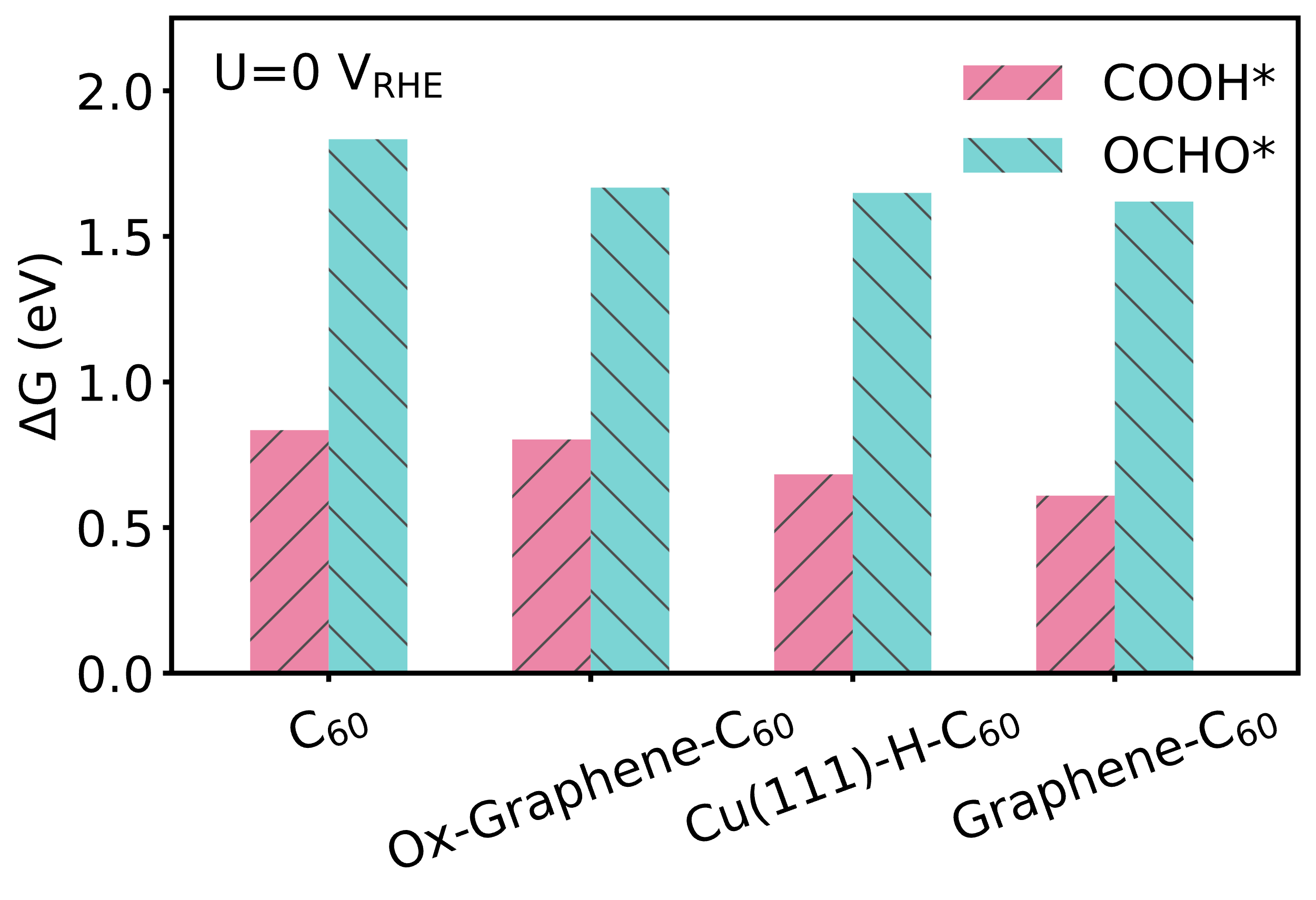
**

**Figure S11.** Free energy diagram of COOH* and OCHO* formation on (a) C_60_, (b) Ox-Graphene-C_60_, (c) Cu(111)-H-C_60_, and (d) Graphene-C_60_ with U = 0 V_RHE_.

**15. Free energy diagrams for COOH-Path with U = 0 V_RHE_**

**Figure S12.** Free energy diagrams considering pH effects of COOH-Path on (a) C_60_, (b) Ox-Graphene-C_60_, (c) Cu(111)-H-C_60_, and (d) Graphene-C_60_ at pH = 1, 7.6, and 14 with U = 0 V_RHE_.

**16.** **Traditional activity volcano of C_60_-based catalysts**

**Figure S13.** Traditional pH-independent activity volcano models of (a) E_CO*_ *vs*. E_COOH*_ and (b) E_HCOOH*_ *vs*. E_OCHO*_ on the C_60_-based catalysts.

**17.** **Scaling relationships of different adsorbates**


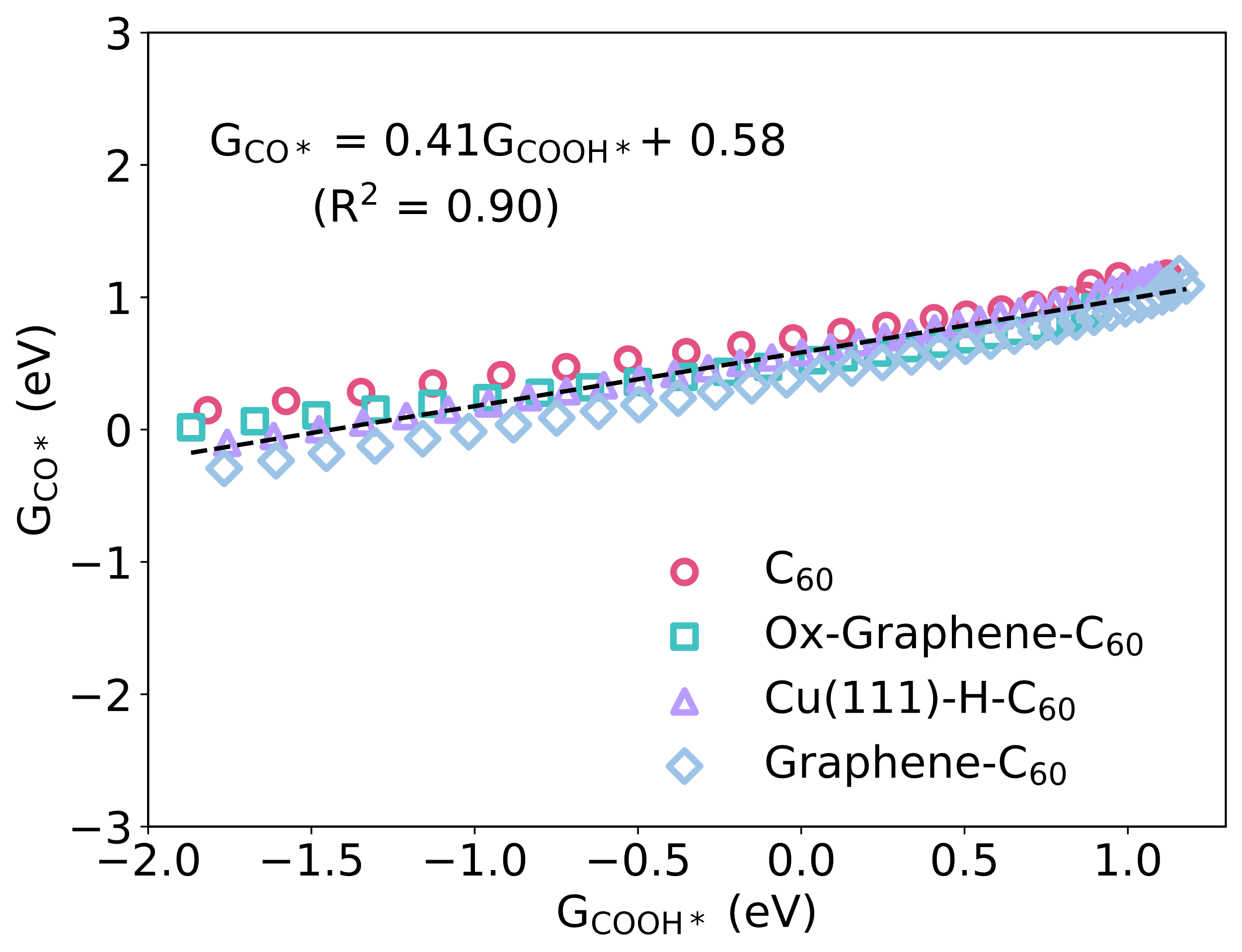


**Figure S14.** Linear scaling relations found in C_60_-based catalysts of COOH-Path under various electric fields.

**18.** **RDS analysis**


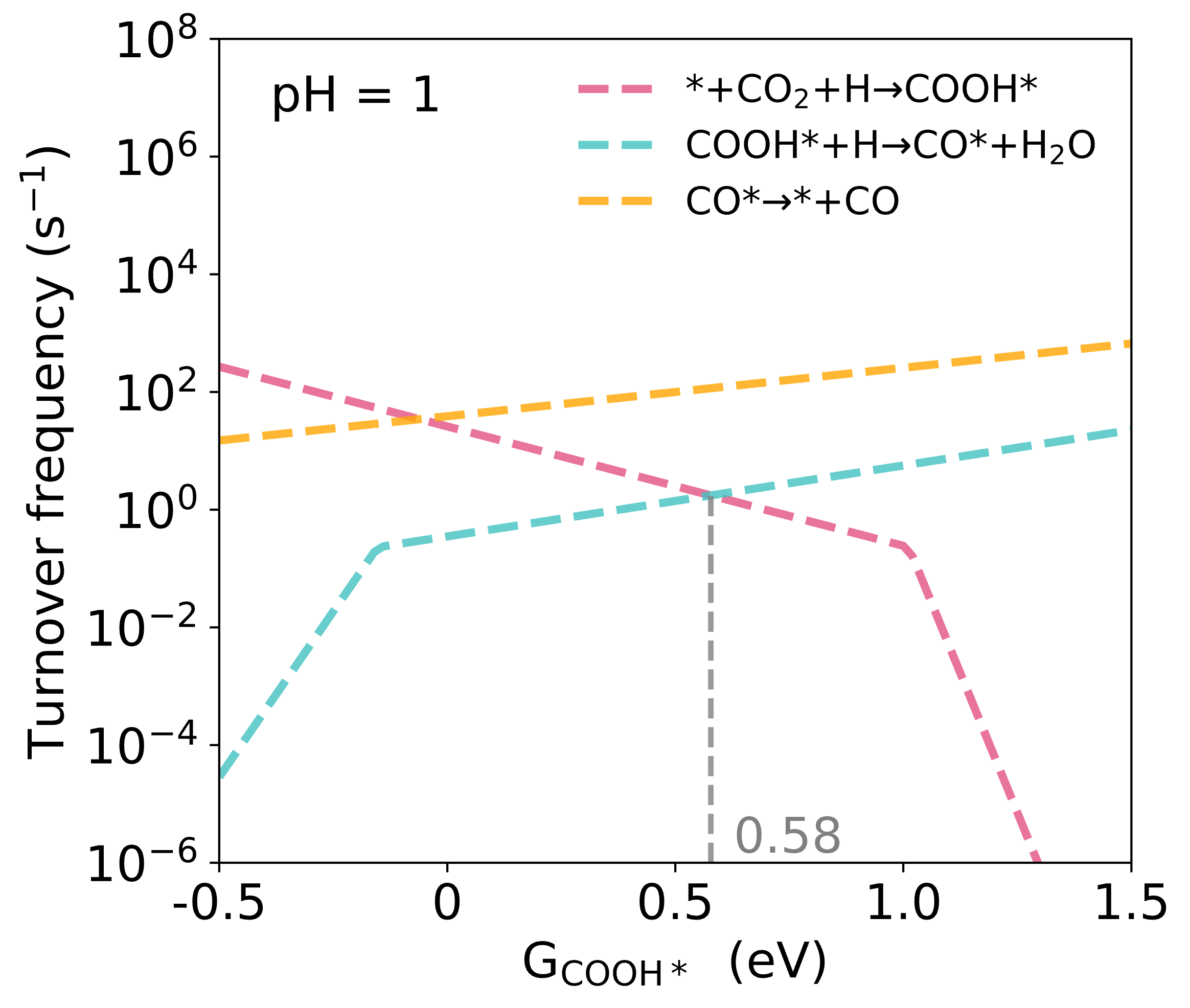


**Figure S15.** RDS analysis of microkinetic CO_2_RR volcano models for C_60_-based catalysts in acidic media.

**19.** **Thermodynamic corrections**

**Table S3.** Summary of the DFT-calculated energies, zero-point energies, heat capacities, and entropic contributions.^7, 65^ Unit: eV

| **Species** | $\text{E}^{\text{DFT}}$ | ***ZPE*** | **∫**$\boldsymbol{C}_{\boldsymbol{p}}\boldsymbol{dT}$ | $\boldsymbol{-TS}$ |
| --- | --- | --- | --- | --- |
| CO_2_ (g) | -22.51 | 0.31 | 0.10 | -0.66 |
| H_2_ (g) | -7.04 | 0.28 | 0.09 | -0.40 |
| HCOOH(*l*) | -29.42 | 0.90 | 0.11 | 0.99 |
| CO(*g*) | -14.59 | 0.13 | 0.09 | -0.61 |
| OCHO* | - | 0.62 | 0.11 | -0.24 |
| COOH* | - | 0.62 | 0.10 | -0.19 |
| CO* | - | 0.19 | 0.08 | -0.16 |

References

(1) Kresse, G.; Furthmüller, J. Efficiency of ab-initio total energy calculations for metals and semiconductors using a plane-wave basis set. *Computational Materials Science* **1996**, *6* (1), 15-50.

(2) Hammer, B.; Hansen, L. B.; Nørskov, J. K. Improved adsorption energetics within density-functional theory using revised Perdew-Burke-Ernzerhof functionals. *Physical Review B* **1999**, *59* (11), 7413-7421.

(3) Hansen, H. A.; Rossmeisl, J.; Nørskov, J. K. Surface Pourbaix diagrams and oxygen reduction activity of Pt, Ag and Ni (111) surfaces studied by DFT. *Phys. Chem. Chem. Phys.* **2008**, *10* (25), 3722-3730.

(4) Vinogradova, O.; Krishnamurthy, D.; Pande, V.; Viswanathan, V. Quantifying confidence in DFT-predicted surface pourbaix diagrams of transition-metal electrode–electrolyte interfaces. *Langmuir* **2018**, *34* (41), 12259-12269.

(5) Valdés, Á.; Qu, Z.-W.; Kroes, G.-J.; Rossmeisl, J.; Nørskov, J. K. Oxidation and photo-oxidation of water on TiO_2_ surface. *J. Phys. Chem. C* **2008**, *112* (26), 9872-9879.

(6) Nørskov, J. K.; Rossmeisl, J.; Logadottir, A.; Lindqvist, L.; Kitchin, J. R.; Bligaard, T.; Jonsson, H. Origin of the overpotential for oxygen reduction at a fuel-cell cathode. *J. Phys. Chem. B* **2004**, *108* (46), 17886-17892.

(7) Chan, K.; Tsai, C.; Hansen, H. A.; Nørskov, J. K. Molybdenum sulfides and selenides as possible electrocatalysts for CO_2_ reduction. *ChemCatChem* **2014**, *6* (7), 1899-1905.

(8) Duan, Z.; Henkelman, G. Theoretical resolution of the exceptional oxygen reduction activity of Au (100) in alkaline media. *ACS Catal.* **2019**, *9* (6), 5567-5573.

(9) Sundararaman, R.; Letchworth-Weaver, K.; Schwarz, K. A. Improving accuracy of electrochemical capacitance and solvation energetics in first-principles calculations. *J. Chem. Phys.* **2018**, *148* (14), 144105.

(10) Hormann, N. G.; Andreussi, O.; Marzari, N. Grand canonical simulations of electrochemical interfaces in implicit solvation models. *J. Chem. Phys.* **2019**, *150* (4), 041730.

(11) Fumagalli, L.; Esfandiar, A.; Fabregas, R.; Hu, S.; Ares, P.; Janardanan, A.; Yang, Q.; Radha, B.; Taniguchi, T.; Watanabe, K.; et al. Anomalously low dielectric constant of confined water. *Science* **2018**, *360* (6395), 1339-1342.

(12) Mathew, K.; Kolluru, V.; Mula, S.; Steinmann, S. N.; Hennig, R. G. Implicit self-consistent electrolyte model in plane-wave density-functional theory. *J. Chem. Phys.* **2019**, *151* (23), 4101.

(13) Mathew, K.; Sundararaman, R.; Letchworth-Weaver, K.; Arias, T.; Hennig, R. G. Implicit solvation model for density-functional study of nanocrystal surfaces and reaction pathways. *J. Chem. Phys.* **2014**, *140* (8), 4106.

(14) Schnur, S.; Groß, A. Properties of metal–water interfaces studied from first principles. *New J. Phys.* **2009**, *11* (12), 125003.

(15) Le, J.; Iannuzzi, M.; Cuesta, A.; Cheng, J. Determining potentials of zero charge of metal electrodes versus the standard hydrogen electrode from density-functional-theory-based molecular dynamics. *Physical Review Letters* **2017**, *119* (1), 016801.

(16) Duan, S.; Xu, X.; Tian, Z.-Q.; Luo, Y. Hybrid molecular dynamics and first-principles study on the work function of a Pt(111) electrode immersed in aqueous solution at room temperature. *Physical Review B* **2012**, *86* (4), 045450.

(17) Sakong, S.; Groß, A. The electric double layer at metal-water interfaces revisited based on a charge polarization scheme. *J. Chem. Phys.* **2018**, *149* (8), 4705.

(18) Grimme, S.; Antony, J.; Ehrlich, S.; Krieg, H. A consistent and accurate ab initio parametrization of density functional dispersion correction (DFT-D) for the 94 elements H-Pu. *J. Chem. Phys.* **2010**, *132* (15), 4101.

(19) Trasatti, S. Structure of the metal/electrolyte solution interface: new data for theory. *Electrochim. Acta* **1991**, *36* (11), 1659-1667.

(20) Hansen, H. A.; Viswanathan, V.; Nørskov, J. K. Unifying kinetic and thermodynamic analysis of 2 e^–^ and 4 e^–^ reduction of oxygen on metal surfaces. *J. Phys. Chem. C* **2014**, *118* (13), 6706-6718.

(21) Kelly, S. R.; Kirk, C.; Chan, K.; Nørskov, J. K. Electric field effects in oxygen reduction kinetics: Rationalizing pH dependence at the Pt(111), Au(111), and Au(100) electrodes. *The Journal of Physical Chemistry C* **2020**, *124* (27), 14581-14591.

(22) Zhu, W.; Liu, S.; Huang, R.; Su, Y.; Huang, K.; He, Z. Enhancing CO_2_ electroreduction to C_2_ products on metal–nitrogen sites by regulating H_2_O dissociation. *ACS Applied Materials & Interfaces* **2024**, *16* (20), 26316-26324.

(23) Fang, L.; Wan, M.; Liu, Y.; Reinhart, B.; Jin, Z.; Yang, M.; Che, F.; Li, T. Revealing structural evolution of single atom catalysts during electrochemical CO_2_ reduction by in situ X-ray absorption spectroscopy. *ACS Materials Letters* **2024**, *6* (8), 3343-3350.

(24) Song, P.; Hu, B.; Zhao, D.; Fu, J.; Su, X.; Feng, W.; Yu, K.; Liu, S.; Zhang, J.; Chen, C. Modulating the asymmetric atomic interface of copper single atoms for efficient CO_2_ electroreduction. *ACS Nano* **2023**, *17* (5), 4619-4628.

(25) Leverett, J.; Tran-Phu, T.; Yuwono, J. A.; Kumar, P.; Kim, C.; Zhai, Q.; Han, C.; Qu, J.; Cairney, J.; Simonov, A. N.; et al. Tuning the coordination structure of Cu-N-C single atom catalysts for simultaneous electrochemical reduction of CO_2_ and NO_3_^–^ to urea. *Advanced Energy Materials* **2022**, *12* (32), 2201500.

(26) Gong, S.; Xiao, X.; Wang, W.; Sam, D. K.; Lu, R.; Xu, Y.; Liu, J.; Wu, C.; Lv, X. Silk fibroin-derived carbon aerogels embedded with copper nanoparticles for efficient electrocatalytic CO_2_-to-CO conversion. *Journal of Colloid and Interface Science* **2021**, *600*, 412-420.

(27) Meng, Z.; Luo, J.; Li, W.; Mirica, K. A. Hierarchical tuning of the performance of electrochemical carbon dioxide reduction using conductive two-dimensional metallophthalocyanine based metal–organic frameworks. *Journal of the American Chemical Society* **2020**, *142* (52), 21656-21669.

(28) Chang, F.; Zhu, K.; Liu, C.; Wei, J.; Yang, S.; Zhang, Q.; Yang, L.; Wang, X.; Bai, Z. Construction of Cu─Ni atomic pair with bimetallic atom-cluster sites for enhanced CO_2_ electroreduction. *Advanced Functional Materials* **2024**, *34* (34), 2400893.

(29) Sun, Z.; Li, C.; Wei, Z.; Zhang, F.; Deng, Z.; Zhou, K.; Wang, Y.; Guo, J.; Yang, J.; Xiang, Z.; et al. Sulfur-bridged asymmetric CuNi bimetallic atom sites for CO_2_ reduction with high efficiency. *Advanced Materials* **2024**, *36* (33), 2404665.

(30) Hao, J.; Zhu, H.; Zhao, Q.; Hao, J.; Lu, S.; Wang, X.; Duan, F.; Du, M. Interatomic electron transfer promotes electroreduction CO_2_-to-CO efficiency over a CuZn diatomic site. *Nano Research* **2023**, *16* (7), 8863-8870.

(31) Zhang, L.; Feng, J.; Liu, S.; Tan, X.; Wu, L.; Jia, S.; Xu, L.; Ma, X.; Song, X.; Ma, J.; et al. Atomically dispersed Ni–Cu catalysts for pH-universal CO_2_ electroreduction. *Advanced Materials* **2023**, *35* (13), 2209590.

(32) Jiao, J.; Yuan, Q.; Tan, M.; Han, X.; Gao, M.; Zhang, C.; Yang, X.; Shi, Z.; Ma, Y.; Xiao, H.; et al. Constructing asymmetric double-atomic sites for synergistic catalysis of electrochemical CO_2_ reduction. *Nature Communications* **2023**, *14* (1), 6164.

(33) Shen, Y.; Zhang, H.; Chen, B.; Zhu, C.; Yu, W.; Yang, J.; Fang, Q.; He, Z.; Sun, T.; Song, S. Mechanistic insight into electron orientation by tailoring Ni–Cu atom-pairs for high-performance CO_2_ electroreduction. *Applied Catalysis B: Environmental* **2023**, *330*, 122654.

(34) Sun, G.; Cao, Y.; Li, D.; Hu, M.; Liang, X.; Wang, Z.; Cai, Z.; Shen, F.; Chen, B.; Zhou, K. Dual-atom Cu_2_/N-doped carbon catalyst for electroreduction of CO_2_ to C_2_H_4_. *Applied Catalysis A: General* **2023**, *651*, 119025.

(35) Wang, J.; Huang, Y.-C.; Wang, Y.; Deng, H.; Shi, Y.; Wei, D.; Li, M.; Dong, C.-L.; Jin, H.; Mao, S. S.; et al. Atomically dispersed metal–nitrogen–carbon catalysts with d-orbital electronic configuration-dependent selectivity for electrochemical CO_2_-to-CO reduction. *ACS Catalysis* **2023**, *13* (4), 2374-2385.

(36) Hursán, D.; Timoshenko, J.; Ortega, E.; Jeon, H. S.; Rüscher, M.; Herzog, A.; Rettenmaier, C.; Chee, S. W.; Martini, A.; Koshy, D.; et al. Reversible structural evolution of metal-nitrogen-doped carbon catalysts during CO_2_ electroreduction: An operando X-ray absorption spectroscopy study. *Advanced Materials* **2024**, *36* (4), 2307809.

(37) Yao, D.; Tang, C.; Zhi, X.; Johannessen, B.; Slattery, A.; Chern, S.; Qiao, S.-Z. Inter-metal interaction with a threshold effect in NiCu dual-atom catalysts for CO_2_ electroreduction. *Advanced Materials* **2023**, *35* (11), 2209386.

(38) Wang, H.; Hao, Y.; Sun, Y.; Pan, J.; Hu, F.; Kai, D.; Peng, S. Size Control of Zn, N-doped carbon supported copper nanoparticles for effective and selective CO_2_ electroreduction. *Catalysis Letters* **2023**, *153* (7), 2115-2124.

(39) Zhu, J.; Xiao, M.; Ren, D.; Gao, R.; Liu, X.; Zhang, Z.; Luo, D.; Xing, W.; Su, D.; Yu, A.; et al. Quasi-covalently coupled Ni–Cu atomic pair for synergistic electroreduction of CO_2_. *Journal of the American Chemical Society* **2022**, *144* (22), 9661-9671.

(40) Hao, J.; Zhuang, Z.; Hao, J.; Wang, C.; Lu, S.; Duan, F.; Xu, F.; Du, M.; Zhu, H. Interatomic electronegativity offset dictates selectivity when catalyzing the CO_2_ reduction reaction. *Advanced Energy Materials* **2022**, *12* (26), 2200579.

(41) Ju, W.; Bagger, A.; Hao, G.-P.; Varela, A. S.; Sinev, I.; Bon, V.; Roldan Cuenya, B.; Kaskel, S.; Rossmeisl, J.; Strasser, P. Understanding activity and selectivity of metal-nitrogen-doped carbon catalysts for electrochemical reduction of CO_2_. *Nature Communications* **2017**, *8* (1), 944.

(42) Wang, D.; Wang, J.; Wang, Z.; Zhang, N.; Zeng, J.; Zhong, H.; Zhang, X. Supported Cu/Ni bimetallic cluster electrocatalysts boost CO_2_ reduction. *Precision Chemistry* **2024**, *2* (3), 96-102.

(43) Luo, M.; Fu, X.; Geng, S.; Li, Z.; Li, M. Efficient electrochemical CO_2_ reduction via CuAg doped CeO_2_. *Fuel* **2023**, *347*, 128470.

(44) Wang, J.; Zhang, G.; Liu, H.; Li, Z.; Wang, L.; Tressel, J.; Chen, S. High-performance electrocatalytic reduction of CO_2_ to CO by ultrathin PdCu alloy nanosheets. *Separation and Purification Technology* **2023**, *320*, 124186.

(45) Wei, Z.; Ding, J.; Duan, X.; Chen, G.-L.; Wu, F.-Y.; Zhang, L.; Yang, X.; Zhang, Q.; He, Q.; Chen, Z.; et al. Enhancing selective electrochemical CO_2_ reduction by In situ constructing tensile-strained Cu catalysts. *ACS Catalysis* **2023**, *13* (7), 4711-4718.

(46) Wei, D.; Wang, Y.; Dong, C.-L.; Zhang, Z.; Wang, X.; Huang, Y.-C.; Shi, Y.; Zhao, X.; Wang, J.; Long, R.; et al. Decrypting the controlled product selectivity over Ag−Cu bimetallic surface alloys for electrochemical CO_2_ reduction. *Angewandte Chemie International Edition* **2023**, *62* (19), e202217369.

(47) Zhou, Q.; Tang, X.; Qiu, S.; Wang, L.; Hao, L.; Yu, Y. Stable CuIn alloy for electrochemical CO_2_ reduction to CO with high-selectivity. *Materials Today Physics* **2023**, *33*, 101050.

(48) Ma, X.; Tian, J.; Wang, M.; Jin, X.; Shen, M.; Zhang, L. Metal–organic framework derived carbon supported Cu–In nanoparticles for highly selective CO_2_ electroreduction to CO. *Catalysis Science & Technology* **2021**, *11* (18), 6096-6102.

(49) Dong, W. J.; Lim, J. W.; Park, J. Y.; Yoo, C. J.; Baek, S.; Cho, W. S.; Kim, W.; Lee, J.-L. Electric-field-driven electrochemical CO_2_ reduction of sharpened Sn/Cu catalysts. *Applied Surface Science* **2021**, *565*, 150460.

(50) Zhang, W.; He, P.; Wang, C.; Ding, T.; Chen, T.; Liu, X.; Cao, L.; Huang, T.; Shen, X.; Usoltsev, O. A.; et al. Operando evidence of Cu^+^ stabilization via a single-atom modifier for CO_2_ electroreduction. *Journal of Materials Chemistry A* **2020**, *8* (48), 25970-25977.

(51) Liu, K.; Ma, M.; Wu, L.; Valenti, M.; Cardenas-Morcoso, D.; Hofmann, J. P.; Bisquert, J.; Gimenez, S.; Smith, W. A. Electronic effects determine the selectivity of planar Au–Cu bimetallic thin films for electrochemical CO_2_ reduction. *ACS Applied Materials & Interfaces* **2019**, *11* (18), 16546-16555.

(52) Wang, C.; Cao, M.; Jiang, X.; Wang, M.; Shen, Y. A catalyst based on copper-cadmium bimetal for electrochemical reduction of CO_2_ to CO with high faradaic efficiency. *Electrochimica Acta* **2018**, *271*, 544-550.

(53) Chen, C.; Huang, H.; Chen, Y.; Zhang, Z.; Fu, H.; Li, H.; Huang, W.-H.; Lai, F.; Zhang, N.; Liu, T. Interface-engineered Cu/Cu_2_In metallic aerogels for efficient electrochemical CO_2_ reduction. *ACS Materials Letters* **2024**, *6* (3), 756-764.

(54) Xie, F.; Wang, Z.; Kao, C.-W.; Lan, J.; Lu, Y.-R.; Tan, Y. Asymmetric local electric field induced by dual heteroatoms on copper boosts efficient CO_2_ reduction over ultrawide potential window. *Angewandte Chemie International Edition* **2024**, *63* (37), e202407661.

(55) Xue, J.; Dong, X.; Liu, C.; Li, J.; Dai, Y.; Xue, W.; Luo, L.; Ji, Y.; Zhang, X.; Li, X.; et al. Turning copper into an efficient and stable CO evolution catalyst beyond noble metals. *Nature Communications* **2024**, *15* (1), 5998.

(56) Sirisomboonchai, S.; Machida, H.; Bao Tran, K. V.; Kawasumi, M.; Norinaga, K. Efficient CO_2_ electrochemical reduction by a robust electrocatalyst fabricated by electrodeposition of indium and zinc over copper foam. *ACS Applied Energy Materials* **2022**, *5* (8), 9846-9857.

(57) Zhang, W.; Zhu, N.; Ding, L.; Hu, Y.; Wu, Z. Efficacious CO_2_ adsorption and activation on Ag nanoparticles/CuO mesoporous nanosheets heterostructure for CO_2_ electroreduction to CO. *Inorganic Chemistry* **2021**, *60* (24), 19356-19364.

(58) Dai, S.; Huang, T.-H.; Liu, W.-I.; Hsu, C.-W.; Lee, S.-W.; Chen, T.-Y.; Wang, Y.-C.; Wang, J.-H.; Wang, K.-W. Enhanced CO_2_ electrochemical reduction performance over Cu@AuCu catalysts at high noble metal utilization efficiency. *Nano Letters* **2021**, *21* (21), 9293-9300.

(59) Luo, W.; Xie, W.; Mutschler, R.; Oveisi, E.; De Gregorio, G. L.; Buonsanti, R.; Züttel, A. Selective and stable electroreduction of CO_2_ to CO at the copper/indium interface. *ACS Catalysis* **2018**, *8* (7), 6571-6581.

(60) Dai, L.; Qin, Q.; Wang, P.; Zhao, X.; Hu, C.; Liu, P.; Qin, R.; Chen, M.; Ou, D.; Xu, C.; et al. Ultrastable atomic copper nanosheets for selective electrochemical reduction of carbon dioxide. *Science Advances* **2017**, *3* (9), e1701069.

(61) Wei, X.; Li, Z.; Jang, H.; Gyu Kim, M.; Liu, S.; Cho, J.; Liu, X.; Qin, Q. Switching Product Selectivity in CO_2_ electroreduction via Cu−S bond length variation. *Angewandte Chemie International Edition* **2024**, *63* (39), e202409206.

(62) Peerlings, M. L. J.; Han, K.; Longo, A.; Helfferich, K. H.; Ghiasi, M.; de Jongh, P. E.; Ngene, P. Synthesis and catalytic performance of bimetallic oxide-derived CuO–ZnO electrocatalysts for CO_2_ reduction. *ACS Catalysis* **2024**, *14* (14), 10701-10711.

(63) Ma, X.; Zhang, Y.; Fan, T.; Wei, D.; Huang, Z.; Zhang, Z.; Zhang, Z.; Dong, Y.; Hong, Q.; Chen, Z.; et al. Facet dopant regulation of Cu_2_O boosts electrocatalytic CO_2_ reduction to formate. *Advanced Functional Materials* **2023**, *33* (16), 2213145.

(64) Chen, J.; Wei, X.; Cai, R.; Ren, J.; Ju, M.; Lu, X.; Long, X.; Yang, S. Composition-tuned surface binding on CuZn-Ni catalysts boosts CO_2_RR selectivity toward CO generation. *ACS Materials Letters* **2022**, *4* (3), 497-504.

(65) Christensen, R.; Hansen, H. A.; Vegge, T. Identifying systematic DFT errors in catalytic reactions. *Catal. Sci. Technol.* **2015**, *5* (11), 4946-4949.
